# A coupled agent-based model for France for simulating adaptation and migration decisions under future coastal flood risk

**DOI:** 10.1038/s41598-023-31351-y

**Published:** 2023-03-13

**Authors:** Lars Tierolf, Toon Haer, W. J. Wouter Botzen, Jens A. de Bruijn, Marijn J. Ton, Lena Reimann, Jeroen C. J. H. Aerts

**Affiliations:** 1 Institute for Environmental Studies, VU University, De Boelelaan 1087, 1081 HV Amsterdam, The Netherlands; 2 Utrecht University School of Economics (U.S.E.), Utrecht University, Utrecht, The Netherlands; 3 International Institute for Applied Systems Analysis (IIASA), Laxenburg, Austria

**Keywords:** Natural hazards, Climate-change impacts, Climate-change adaptation

## Abstract

In this study, we couple an integrated flood damage and agent-based model (ABM) with a gravity model of internal migration and a flood risk module (DYNAMO-M) to project household adaptation and migration decisions under increasing coastal flood risk in France. We ground the agent decision rules in a framework of subjective expected utility theory. This method addresses agent’s bounded rationality related to risk perception and risk aversion and simulates the impact of push, pull, and mooring factors on migration and adaptation decisions. The agents are parameterized using subnational statistics, and the model is calibrated using a household survey on adaptation uptake. Subsequently, the model simulates household adaptation and migration based on increasing coastal flood damage from 2015 until 2080. A medium population growth scenario is used to simulate future population development, and sea level rise (SLR) is assessed for different climate scenarios. The results indicate that SLR can drive migration exceeding 8000 and 10,000 coastal inhabitants for 2080 under the Representative Concentration Pathways 4.5 and 8.5, respectively. Although household adaptation to flood risk strongly impacts projected annual flood damage, its impact on migration decisions is small and falls within the 90% confidence interval of model runs. Projections of coastal migration under SLR are most sensitive to migration costs and coastal flood protection standards, highlighting the need for better characterization of both in modeling exercises. The modeling framework demonstrated in this study can be upscaled to the global scale and function as a platform for a more integrated assessment of SLR-induced migration.

## Introduction

The rise in global relative sea levels is accelerating, increasing the probability and severity of coastal flooding for exposed populations^1–3^. Simultaneously, rapid urbanization and socioeconomic development increase exposed assets and people in coastal areas worldwide^4^. Studies estimate that, depending on the sea level rise (SLR) and population change scenario, up to an estimated 260 million people could inhabit the coastal 1/100-year flood zone in 2100, more than a twofold increase compared to 2000^5^. Much of this development is occurring in regions where the implementation of coastal protection infrastructure is low or even absent^6,7^. Simultaneously, SLR and associated impacts such as increased flood events, salinization, and erosion make living in coastal areas more challenging, potentially leading to migration and adaptation^8^.

SLR may prompt inhabitants and governments to adapt to increasing flood hazards, for example, through elevating properties or constructing sea walls^9^. However, governments must choose which areas will be protected, as governmental investments in coastal protection will not be economically feasible for the entire coastline^10^. If protection levels remain insufficient, SLR can be a major driver of migration away from coastal zones for those who can afford it^7,8,11^, while impoverished communities may become trapped in hazardous locations^12^. Those who find ways to migrate often find themselves in less desirable locations or different vulnerable floodplains^13^. Therefore, policymakers will probably need to resort to planned retreat once coastal protection becomes economically unviable^14–17^. Local adaptation strategies and migration policies can be prioritized by identifying areas where individuals can stay and adapt despite SLR and areas where migration will be the inevitable outcome^18^.

A growing body of literature investigates migration through modeling studies^8,19,20^. These studies depict migration as a complex process in which push factors act on sending areas and pull factors on destination areas. The gravity model of migration is a commonly applied model to assess the effect of push and pull factors such as employment, income, and social network on migration flows^21,22^. Resembling Newton’s law of gravity, this model assumes that the intensity of migration flows between locations depends on their population sizes and that these flows are negatively correlated with distance^23,24^. Explanatory push and pull factors, such as unemployment rates and income differentials, can be included in the gravity equation to estimate their relative effect on migration flows^25^. For example, recent studies applied a gravity-based model to project internal migration driven by SLR and other environmental factors, such as water scarcity and crop productivity^26,27^. In this research, a gravity model is operationalized by calculating the population potential for each grid cell based on a distance-density gradient and location-specific push and pull factors, additionally accounting for areas that will be permanently inundated under different SLR scenarios. The results show large differences in internal migration flows under different global socioeconomic change scenarios, highlighting the potential of gravity models to capture migration flows.

However, in current gravity modeling approaches, flood adaptation is rarely addressed, resulting in a potential under- or overestimation of migration flows from coastal areas^8^. Furthermore, gravity-based modeling approaches fail to capture differences between individuals, such as individual preferences and experiences, major determinants of decisions to stay or migrate away from harm^8^. Generally, people exhibit bounded rationality and base their behavioral choices on their experiences and the limited information available to them^28^. For example, people residing in hazardous coastal floodplains may choose not to adapt or migrate simply because they do not perceive themselves as at risk of flooding, while the objective information from empirical data shows that they live in a flood zone. This perception may change, and underestimations of real flood risk may become overestimations of risk after experiencing a severe flooding event^29^. Furthermore, both flood risk and the perception of flood risk are dynamic; for example, adaptation by individuals or governments may reduce flood risk. Moreover, socioeconomic characteristics, such as wealth and income, can inhibit people from adopting property-level adaptation measures if they are considered unaffordable to the household^30^, while attachment to place often results in voluntary immobility in areas exposed to environmental hazards^31^.

Agent-based models (ABMs) provide researchers with a means to explicitly model these interpersonal differences and their influence on migration decisions^32^. For example, in the Dynamic adaptation model (DYNAMO)^33–35^, agents can be individuals or households but also governments or insurers. Interactions between these agents and feedback loops between environmental hazards and adaptation to flooding (including migration) produce emergent behavior that cannot be captured by statistical analysis of migration flows, as conducted with the gravity model. By embedding the ABM decision rules of households and governments in behavioral theory, ABMs can build on findings from economic and social sciences, contributing to scientific discussion of climate-induced migration^20,36,37^.

In this study, we develop a new simulation model, DYNAMO-M, which (1) integrates a migration module into an ABM^34,35^, (2) couples the ABM to a gravity model to simulate internal migration flows outside and toward coastal areas, and (3) simulates flood adaptation driven by coastal flooding as simulated with a flood risk model of yearly coastal flood risk (see Methods). Flood risk is estimated per household, using household-specific depth-damage curves and flood inundation levels under different SLR scenarios^38^. As recommended by Klabunde and Willekens^32^, household decision rules in the ABM are grounded in subjective expected utility theory^39^. Here, we apply DYNAMO-M for France, capturing differences in household characteristics using subnational statistics and calibrating household behavior on empirical survey data. The model is applied to simulate adaptation uptake and internal migration flows under different SLR scenarios and future population growth.

## Case study: France

We apply the ABM developed in this study to France, where population development and rising sea levels are projected to increase the population in the coastal 1/100-year flood zone from 1.6 million in 2000 to 2.33 million in 2060^40^. This exposure to coastal floods became apparent to the public in 2010, when storm Xynthia hit the Atlantic coast. The combination of storm surge and high tide resulted in 47 casualties, and the economic damage was estimated at 2.5 billion euros^41^. The surge height and associated flooding exceeded the 1 in 100-year water levels, based on historical data^42^. A sea level rise of 50 cm in the area affected by Xynthia could change the return period of the current 1 in 100-year flood into a 1 in 10-year flood^43^.

France has no national standard for flood protection levels^44^. Levees typically protect agricultural areas against 1 in 5 to 1 in 10-year events and populated areas against 1 in 20 to 1 in 100-year events^45^. When Xynthia struck in 2010, most coastal defenses—many constructed in the Napoleonic era—were in a poor state of repair^44,46^. Besides structural measures to reduce flood hazards, the French government aims to reduce exposure to flooding through zoning laws. The *Loi Littoral* (Littoral Law) of 1986 inhibits residential development within 100 m of the shoreline. Risk management on the city scale is based on a *Plan de Prévention des Risques-Littoraux* (Littoral Risk Prevention Plan; L-RPP), prepared by the central government. The L-RPP identifies the limits of the floodplain and maps hazard zones, each associated with its own building restrictions^47,48^.

Although the state is legally responsible for a flood risk management strategy complying with the EU Floods Directive, information gathering and practical undertaking of the L-RPP is often left to local authorities and stakeholders operating with a limited budget^44,49^. The approval procedure of L-RPPs is often slow, and inundation maps used to define hazard zones are often low-quality^50^. Due to high coastal urbanization levels, designating extremely hazardous areas (“solidarity zones”) often requires the state to buy out property owners. This process is often met with fierce community resistance^51^. Following the L-RPP, Mercier and Chadenas^51^ support the need for property-level flood adaptation in the (future) coastal floodplain, as many of these areas have seen substantial residential development in the past. However, L-RPPs often fail to stimulate the implementation of damage reduction measures, such as pumps or anti-backflow valves, that prevent floodwaters from entering buildings^48^. Hence, installing flood damage mitigation measures and relocation away from floodplains is largely an individual decision in France.

## Results

### Simulations of the exposed population, migration, and residential flood risk

In this study, we developed a coupled calibrated agent-based gravity model (see Supplementary information S2.1) that simulates household migration and adaptation under different SLR scenarios and applied this model to France. In contrast to traditional coastal flood risk assessments, the model considers household characteristics, such as place attachment, income, and risk perception, as well as push and pull factors, such as flood risk and income differentials (see Methods). Our results indicate that including adaptation and migration lowers flood risk expressed as the expected annual damage (EAD; EUR/year) compared to model runs that neglect adaptation and migration.

**Figure 1:**
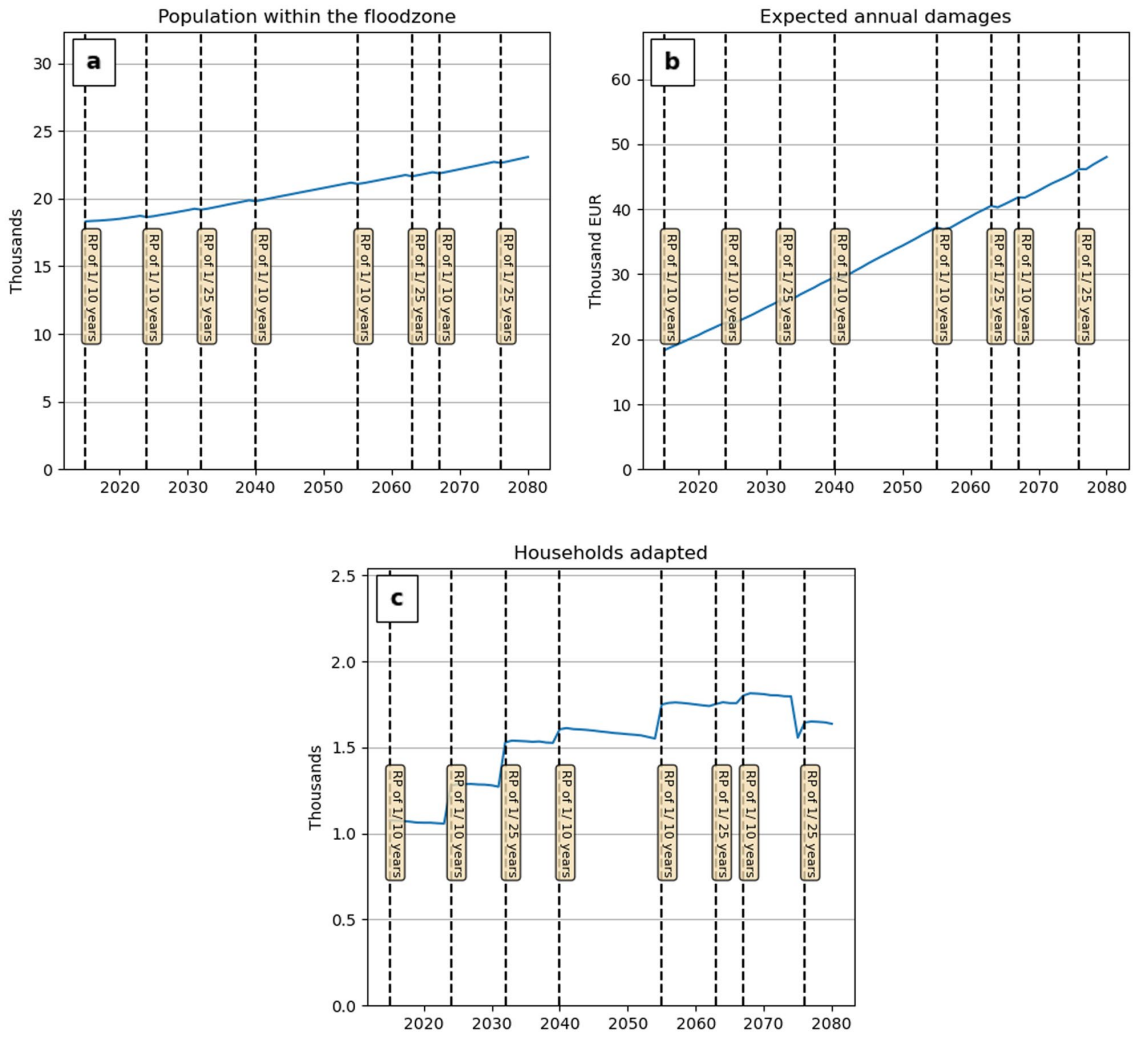
Projected population change and EAD for the 1/100-year flood zone of Vendée under RCP 4.5. Dashed vertical lines indicate stochastic flood events; labels show the exceedance probability of the event.

**Figure 2:**
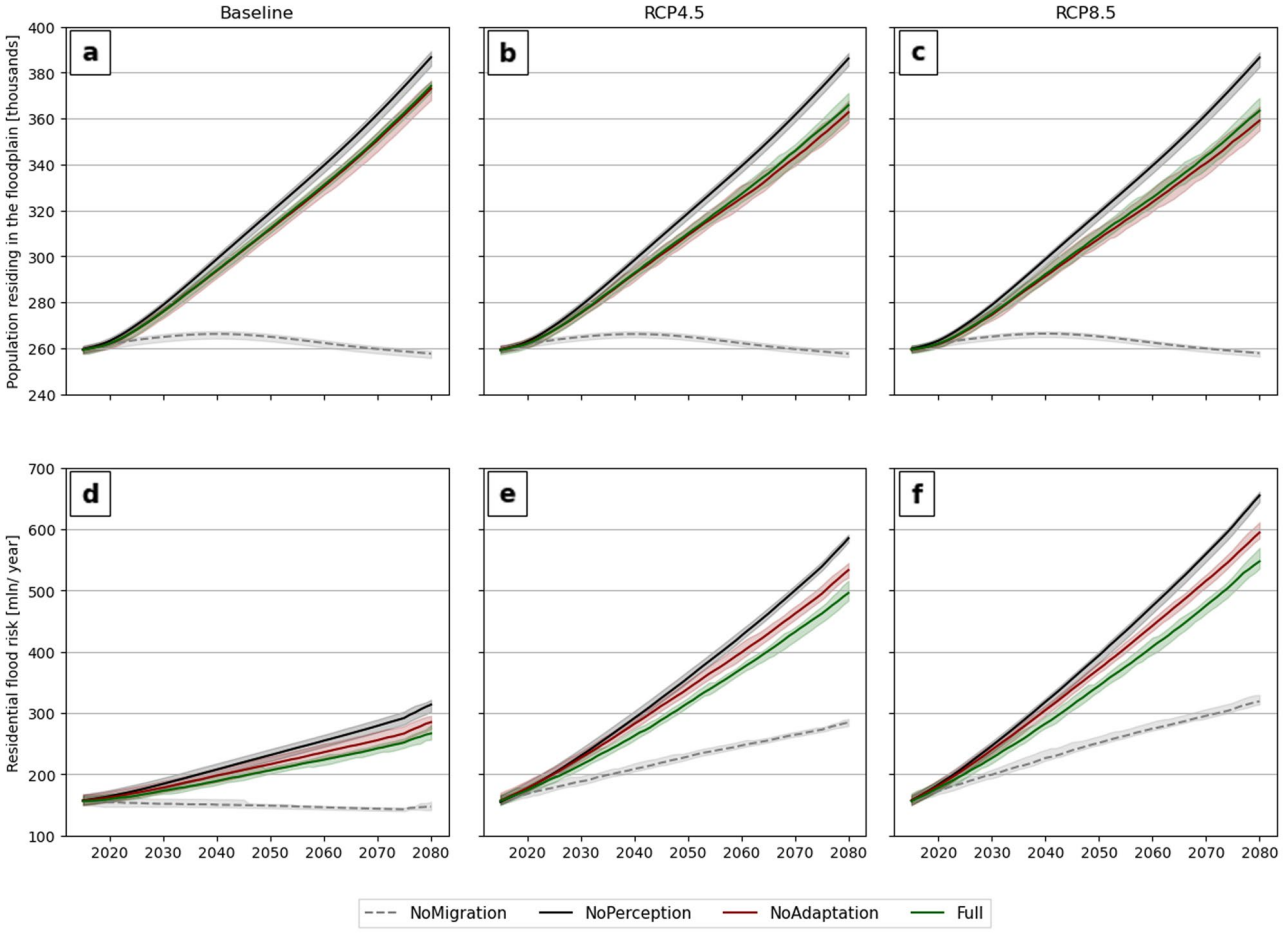
Simulated population (upper three panels) and EAD (lower three panels) in the coastal flood zone for four behavioral settings (NoMigration, NoPerception, NoAdaptation, and Full) and three climate scenarios: Baseline (left panels), RCP 4.5, and RCP 8.5 (panels at center and right, respectively). Shading indicates the lower and upper bounds of 50 repetitive model runs per behavioral setting. Solid lines indicate the means of these runs. Each model run is initiated with a spin-up period of 15 years in which we apply the Full model setting.

To illustrate the model dynamics, we first show the results for a single run for Vendée under RCP 4.5 (Fig. 1) and then discuss the results based on multiple runs on the national scale (Fig. 2). The experience of flooding drives migration and adaptation decisions through increased risk perceptions, illustrated here by a small dip in population and a larger decrease in the EAD immediately after a flood event (Fig. 1a, b), The EAD decreases after each flood event (Fig. 1b), as overestimations of flood risk prompt households to migrate or implement dry flood-proofing measures (Fig. 1c). However, this reduction in the EAD is rapidly offset by an increase in the exposed population due to coastward migration and SLR (Fig. 1a). Two other individual model runs including migration flows are described in more detail in Supplementary information S3.1.

Figure 2 shows the development of the total population residing in the coastal 1/100-year flood zone and the EAD over time based on 50 calibrated model runs. We show the nationally aggregated results for three SLR scenarios (Baseline, RCP 4.5, and RCP 8.5) combined with four behavioral settings:

1. *NoMigration*: natural population growth, no migration, including adaptation behavior. The population only changes due to natural population growth and decline projected under the World Population Prospects scenarios^52^. Households have a dynamic perception of flood risk and may implement dry flood-proofing measures (see Methods).
2. *NoPerception:* natural population growth, with economic migration, no migration, and adaptation based on (increasing) flood risk because households have no (dynamic) risk perception and permanently underestimate flood hazard (*β* = 0; Methods Eq. 5).
3. *NoAdaptation*: natural population growth with economic migration and migration due to flood risk but without adaptation. Households have a dynamic perception of flood risk and may choose to migrate or stay but have no options to implement adaptation measures.
4. *Full:* natural population growth, including all migration and adaptation. Here, we simulate household adaptation, inland and coastal migration, and dynamic risk perceptions in response to flood experience.

The model is run for these four settings to assess the effect of coastward migration on population development (*NoMigration*) and the effects of flood risk perception and local adaptation on migration decisions (*NoPerceptions* and *NoAdaptation*). In the model setting of no coastward migration (*NoMigration,* Fig. 2a–c), the total population residing in the coastal flood zone peaks around 2045 and declines until 2080, indicating that without coastward migration, the population within the 1/100-year flood zone will decrease over time following the World Population Prospects projections^52^. Under setting *NoPerception,* the total population residing in the 1 in 100-year flood zone increases from 259,552 inhabitants in 2015 to ~ 386,000 inhabitants in 2080 under all scenarios (Fig. 2a–c). In this behavioral setting, migration is only driven by income differences and coastal amenities, as the coastal flood hazard is not perceived by the household agents (*β* = 0). When including dynamic risk perceptions, migration, and household adaptation (*Full*), this total population increases to 374,033 under the baseline scenario (Fig. 2a). This result shows an effect of flood risk on migration decisions in the coastal flood zone, leading to more households choosing to migrate inland compared to migration driven only by income differentials (3.2% of the total population under the baseline scenario until 2080).

Under the future climate scenarios (RCP 4.5 and 8.5), the total coastal population in the *Full* setting increases to 365,721 and 363,215 in 2080, respectively (Fig. 2b, c). SLR thus results in fewer people being allocated to the coast. Since this difference is mainly due to increased out-migration rates, we suggest that SLR results in an additional 8,345 coastal emigrants under RCP 4.5 and 10,934 coastal migrants under RCP 8.5 compared to *Full-baseline*. This corresponds to net migration rates of 2.23% and 2.99% under RCP 4.5 and 8.5, respectively. When local adaptation is not considered as a strategy by the households (*NoAdaptation*), SLR drives 10,414 (2.8%) and 13,263 (3.7%) coastal emigrants under RCP 4.5 and 8.5 (Fig. 1b, c). These projections show a preference for coastal inhabitants to adapt and stay compared to migrating under current model settings. However, these differences fall within the 5th and 95th percentiles of model runs.

EAD increases from EUR 156 million in 2015 to EUR 313 million in 2080 in *NoPerception* under the baseline scenario (Fig. 2d). Under this scenario, exposure change due to migration and natural population growth is the only driver of increasing coastal flood risk. Since households perceive no flood risk under this behavioral setting, household-level adaptation is not considered in these model runs. When only including household migration (*NoAdaptation*) in the model runs, the EAD increases to EUR 286 million in 2080 under the baseline scenario (Fig. 2d). When adaptation is simulated as an alternative strategy to migration (*Full*), the EAD increases to EUR 267 million (Fig. 2d). Simulating household migration and adaptation in response to flood risk reduces the EAD in 2080 from 313 million to EUR 267 million under the baseline scenario.

Our simulations show a large effect of SLR on EAD. Without simulating household adaptation and migration driven by perceptions of flood risk (*NoPerception)*, the EAD increases from EUR 156 million in 2015 to EUR 585 million and EUR 655 million in 2080 under RCP 4.5 and 8.5, respectively (Fig. 2e, f). Under the *Full* setting, the EAD increases to EUR 497 million and EUR 549 million under RCP 4.5 and 8.5, respectively (Fig. 2e, f). Simulating household decision-making under flood risk reduces the EAD to EUR 88 million (− 15%) under RCP 4.5 and EUR 106 million (− 16%) under RCP 8.5.

The results for the individual departments are presented in Supplementary Table S7.

### Model comparison of flows between the gravity model and ABM

**Figure 3:**
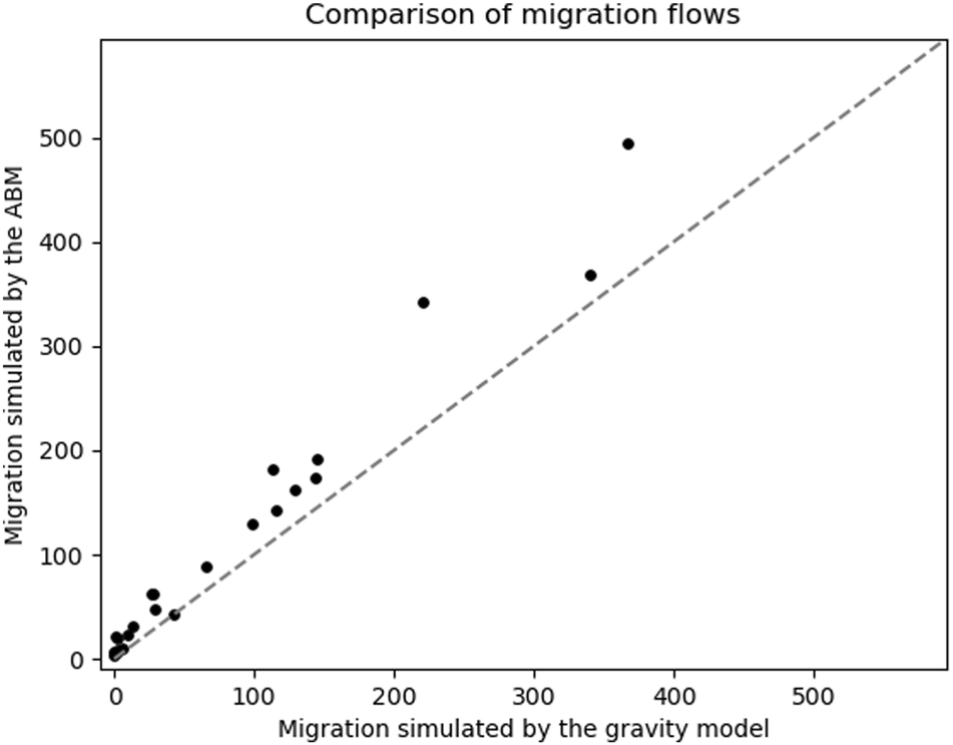
Flow comparison of outmigration via the gravity model and ABM across all coastal nodes. Each dot represents a coastal node, we show the average annual outmigration over a period of 10 years.

To assess whether the migration flows simulated by the ABM are consistent with migration flows produced by the gravity model, we provide a direct comparison of outmigration from coastal nodes simulated by both models. We run the model twice for a period of 10 years. First, we apply the ABM to simulate household decisions under the *full* model setting, considering a scenario of no SLR to minimize the effect of coastal flood risk on migration flows in the ABM. Then, we run the model treating all nodes (coastal and inland) as inland nodes. The resulting flows out of each coastal node under both approaches are shown in Fig. 3. This result shows although the ABM produces slightly more outmigration than the gravity mode in most coastal nodes, both models generate similarly scaled results. We argue that the increased outmigration can at least be partly attributed to flood risk driven migration, which is not included in the gravity model used, and which in normal simulations is only used for inland regions. A table showing the results for the individual nodes is provided in Supplementary Table S7.

### Sensitivity analysis

A one-at-a-time sensitivity analysis is performed to assess the model robustness to uncertainties in migration costs, flood protection standards (FPS), and conversion of migration intention to behavior on model projections. We choose these variables for our analysis due to the wide range of values found in the literature (see Methods) and a lack of data specific to the case study area. The results of the sensitivity analysis are presented in Supplementary information S2.4.

The migration projections demonstrated to be most sensitive to changes in FPS, migration costs and the factor converting migration intentions to migration behavior. The impact of FPS on migration decisions is twofold: they (1) decrease the frequency whereby households experience flooding, lowering the total number of households with heightened risk perceptions, and (2) affect the expected utility of staying by reducing the probability of a flood event. The effect of migration costs on migration decisions is straightforward. Raising the costs requires a higher return of migration, whereas a low migration cost is rapidly offset by an increased net present value after the initial investment cost of the move. This lower migration cost also resulted in more outmigration driven by income differentials, lowering the relative contribution of SLR to migration flows. Since fixed migration costs combine direct monetary and indirectly monetized psychological costs^53,54^, stated preference survey data, such as choice experiments^55^, could help estimate these costs for households affected by SLR.

The factor converting migration intentions to migration behavior also influences the amount of SLR induced migrants. Longitudinal surveys on the effect of migration intentions on actual migration behavior after the experience of a flood event will benefit the validation of these simulated dynamics^56^.

We calibrate risk perception on the observed implementation rate of dry floodproofing measure and test the effect of different risk perception settings on simulated migration. The impact of risk perceptions on SLR induced migration is straightforward: a greater overestimation of flood risk resulted in more SLR driven migration. Removing household overestimation of risk by setting the peak risk perception at 1 resulted in less migration out of the flood zone but resulted in an adaptation uptake that did not match the survey data.

## Discussion

A growing number of studies aim to project the impact of SLR on the coastal EAD and migration^19,20,57^. Most studies assume people only migrate once they are permanently inundated or do not consider migration outcomes under different climate scenarios^20,58^. We contribute to the literature by providing spatially explicit projections of household adaptation and migration under different climate scenarios, while considering individual household characteristics, such as income, wealth, and risk perception, with push (flood hazard) and pull (income differentials) factors influencing these decisions^8^. The modeling framework presented in this study thereby offers a more nuanced approach to simulating coastal flood risk and SLR-induced migration.

We project the population in the coastal 1/100-year flood zone to increase by 43% in 2080 under a scenario of no SLR and by 41% and 40% under RCP 4.5 and 8.5, respectively. These future projections are low compared to existing research findings that neglect SLR-induced migration. Applying the same medium population growth scenario of the World Population Prospects^52^, Neumann et al.^40^ project that a 43% population increase in the 1/100-year flood zone in France will be reached in 2060. Vousdoukas et al.^57^ project even more coastal population growth in France for the twenty-first century, stating that the population exposed to coastal flooding could increase from 3000 people in 2000 to 377,000 in 2100. This difference also influences the projected EAD. Where we project an increase in the EAD from EUR 156 million in 2015 to EUR 549 million in 2080 (RCP 8.5), Vousdoukas et al.^57^ project an increase from EUR 100 million in 2000 to EUR 203 billion in 2100 (RCP 8.5).

Our lower population growth projections for the coastal flood zone indicate that our model results could be interpreted as lower bound estimations. We found that SLR under RCP 4.5 and 8.5 drove 8345 and 10,934 people to migrate from the flood zone, respectively. This is much lower than the number of migrants projected by Lincke and Hinkel^59^, who, without a framework of push, pull, and mooring factors, project the number of SLR-induced migrants in France to range between 54,006 and 386,274 people in the twenty-first century. Our sensitivity analysis showed that projections of SLR-induced migrants depend on mooring factors (captured in fixed migration costs), indicating that populations could persist in locations of high expected damage if the initial investment in migration is not offset by expected utility gains following the move (Supplementary Table S8). Another factor explaining this difference could be the FPS applied in the study. We found that raising or lowering the FPS strongly affects migration decisions in the coastal flood zone. The assumption that FPS remain constant over time may result in overestimations of SLR-induced migration, as the economic motivation to raise FPS will probably increase due to increased coastal flood hazard and human development in coastal areas^60^.

Another limitation arose concerning the lack of survey data to calibrate the migration decisions of households affected by long-term SLR. Surveys and choice experiments could provide modelers with information on household decision-making under hypothetical future flood scenarios, which could then be used to validate the simulated adaptation and migration behavior under SLR scenarios^61^.

## Conclusion and recommendations

This study presents a novel modeling approach (DYNAMO-M) to project future coastal migration and adaptation under different SLR scenarios. A gravity model of migration is coupled to an ABM simulating household adaptation and migration behavior in the coastal 1/100-year flood zone. We calibrate and validate the model using empirical data and run the model to project coastal adaptation and migration in France until 2080. SLR may drive more than 12,000 and 19,000 coastal emigrants for 2080 under RCP 4.5 and 8.5, respectively. The EAD increases from EUR 200 million in 2015 to EUR 626 million and EUR 684 million under RCP 4.5 and 8.5, respectively. Although households have a slight preference for implementing adaptation measures compared to migration, the effect of including local adaptation as a strategy fell within the 90% confidence interval of model runs. Migration decisions were demonstrated to be most sensitive to fixed migration costs and coastal protection standards (FPS), highlighting the need for better characterization of both in studies of SLR-induced migration. Moreover, raising FPS may serve as an extremely efficient policy option to reduce SLR-induced migration, allowing governments to keep coastal regions attractive for habitation in the future. However, the potential levee effect of this strategy should be considered in future research, as the coastal population exposed to flooding will continue to increase under SLR. This effect refers to the feature that governmental flood protection can reduce the incentive for autonomous adaptation by local households. Because of these investments, people in the flood zone feel more safe, and are less inclined to migrate^62^. Furthermore, future research could also investigate the influence of time preferences related to adaptation investments. Time preferences vary amongst individuals and may, like risk perceptions, change in response to experience with disaster^63^.

Fixed migration costs capture both direct monetary and indirectly monetized psychological costs associated with leaving a coastal community and could be better characterized in future research using stated preference survey data, such as choice experiments^55^. Choice experiments have been applied in various studies of managed retreat to assess a willingness to pay, and could benefit the calibration and validation of models of autonomous household migration out of the flood zone^64,65^. Furthermore, development of place attachment in destination areas could be an interesting addition to the model. Models could include a process of place detachment, in which communities intentionally loosen up their current place attachment and form an attachment elsewhere^66^. The same applies to income mobility and education. Education opportunities establish income mobility, and access to education gives people the capacity to migrate that would otherwise be immobile^67^. Including such developments in this ABM of migration under SLR would be an interesting addition.

Migration is a complex process, and many factors play a role. Developments in inverse generative social science (IGSS) could help increase our understanding of the decision-making processes of households currently affected by SLR. In IGSS, machine learning and artificial intelligence techniques are applied to infer plausible model structures and agent architectures that could result in the observed emergent behavior on the macro level^68^. In combination with big data approaches, such as nighttime light analysis to infer displacement after flood events^69^, IGSS could yield interesting results when constructing decision rules for ABMs of SLR induced migration.

SLR not only increases coastal flood risk for exposed populations but also affects migration decisions through exacerbating coastal erosion and saltwater intrusion^8^. Although efforts have been made to capture the impact of both coastal processes on future migration, the migration decision simulated in these studies is either simplified^70^ or region-specific^71^. In future research, we aim to upscale the modeling framework proposed in this study to provide global projections of SLR-induced migration. In such a global model, it is important to address the impact of soil salinization and coastal erosion on the expected utility of migration and adaptation. By using survey data to calibrate and validate agent decision-making, DYNAMO-M could function as a platform for developing a more integrated assessment model of SLR-induced migration and adaptation.

## Methods

### Model setup

**Figure 4:**
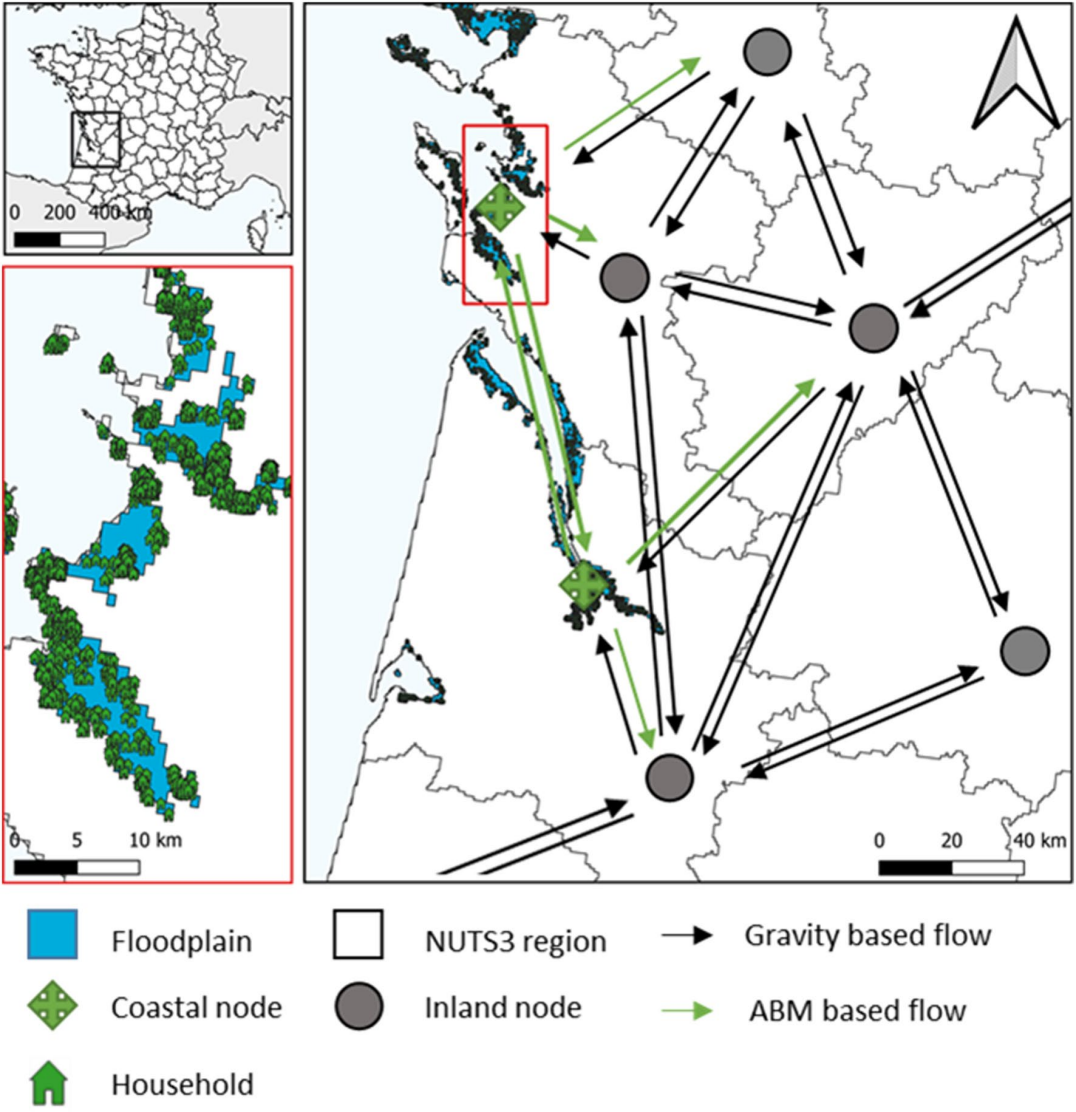
Schematic overview of the modeling framework for a selection of departments in France. We model the adaptation and migration decisions of spatially explicit households residing in the coastal 1/100-year flood zone of 2080 in all of France based on expected utility theory (Floodplain). Migration flows between all inland nodes and toward coastal nodes (representing the households in flood zones) are simulated using a gravity-based model. Households moving from inland nodes toward the coast are made spatially explicit based on a suitability map generated using travel time to the urban area and distance to the coast. Note that, for clarity, only a part of France is shown, and not all linking arrows are drawn in this figure. This figure was generated using QGIS 3.22.13 (QGIS Association: https://qgis.org/).

In this study, we couple an ABM (DYNAMO^35^) with a gravity model. The model contains a flood risk module using household-specific depth-damage curves and dynamically generates flood events based on flood hazard maps for various return periods under different climate change scenarios^38,72^. We code the model using the honeybees ABM environment^73^ and simulate adaptation and migration decisions with a yearly time step. Figure 4 presents a schematic overview of the coupled model developed in this study. The case study area in France has been divided into departments (NUTS-3 administrative units). All inland departments are represented by an inland node. Each coastal department is split into two zones; an inland zone and a flood zone based on the projected 1 in 100-year flood map for 2080 are used under the RCP 8.5 emission scenario^74,75^. The inland zone of the coastal department is represented by an inland node (resembling inland departments) and the flood zone by a coastal node. We then generate household agents only for the 1/100-year flood zone using a gridded population map for 2015^76^ and group individuals into households with an average household size of 3.5 people using a uniform distribution ranging from 1 to 6. Like coastal nodes, inland nodes have information on income and population size, but households in the inland zones are only simulated as an aggregate population to reduce computational demand. A full model description following the Overview, Design concepts and Details plus Decisions (ODD + D) protocol is included in the Supplementary Information^77^.

Migration flows are simulated in three parts:

1. *Migration flows from the coastal flood zone nodes to all other nodes* (coastal flood zone decisions; ABM): These decisions within the coastal flood zone are simulated using the ABM and the discounted expected utility (DEU) theory. Households decide to migrate once the utility of staying in the coastal flood zone (with and without adaption) is lower than the DEU of migration to other coastal and inland nodes.
2. *Migration from inland to coastal nodes and between inland nodes* (gravity model): We apply a calibrated gravity model to simulate migration flows between inland nodes and from inland nodes to coastal nodes.
3. *Distribution of coastal in-migration from all nodes* (coastal in-migration): After simulating all migration flows, the households moving into a coastal node are spatially distributed in the 1/100-year coastal flood zone based on flood risk (mediated by the agent’s flood risk perception) and coastal amenity value. We now discuss each of the migration steps in more detail.

### Coastal flood zone decisions (ABM)

In the coastal flood zone, we build on the DYNAMO model^34,35^, where households make bounded rational decisions based on DEU theory^39^. This theory has been applied in various ABMs simulating population mobility and household adaptation to flooding^20,35,58,62^. One key benefit of DEU is that it enables direct weighing of households’ adaptation options (including migration), while accounting for different risk perceptions and risk preference changes over time based on experiences with flooding and trust in authorities’ protection^78–81^.

In each time step representing 1 year, each household agent calculates and compares the DEU of the following:

1. Doing nothing (Eq. 1)
2. Implementing dry flood-proofing measures (Eq. 2)
3. Migrating to node *y* (Eq. 3)

The agent executes the strategy yielding the highest utility within its budget constraints (see *Budget constraints*). The formulas for calculating the DEU of each strategy are as follows:

1 $$DE{U}_{1}= {\int }_{{p}_{i}}^{{p}_{I}}{\beta }_{t} *{p}_{i}*U \left(\sum_{t=0}^{T}\frac{{W}_{x}+{A}_{x}+{Inc}_{x}-{D}_{x,t,i} }{{\left(1+r\right)}^{t}}\right)dp$$

2 $$DE{U}_{2}= {\int }_{{p}_{i}}^{{p}_{I}}{\beta }_{t} *{p}_{i}*U \left(\sum_{t=0}^{T}\frac{{W}_{x}+{A}_{x}+{Inc}_{x}-{D}_{x,t,i}^{adapt} -{C}_{t}^{adapt}}{{\left(1+r\right)}^{t}}\right)dp$$

3 $$DE{U}_{3}=U\left(\sum_{t=0}^{T}\frac{{W}_{y}+{A}_{y}+{Inc}_{y}-{C}_{y}^{migration}}{{\left(1+r\right)}^{t}}\right)$$

Utility is a function of household wealth (*W*_*x*_), the amenity value of the current household location, *A*_*x*_, current household income *I*_*x*_, expected damage *D* per event *i*, and adaptation costs *C*^*adapt*^. Additional variables for calculating the DEU of migration are prospected income *Inc* and wealth *W* in node *y,* amenity values *A*_*y*_ in node *y,* and migration costs C^migration^ to node *y*.

*Discounting and risk aversion:* A time discounting factor *r* of 3.2% specific to France is applied over a time horizon of 15 years, representing the number of years a homeowner on average stays in his or her home. We assume a general utility function $U\left(x\right)$ as a function of relative risk aversion *σ* (Eq. 4). The model is run with slightly risk-averse households (*σ* = 1). When *σ* = 1, the function $U\left(x\right)=\mathrm{ln}(x)$ is used.

4 $$U\left(x\right)=\frac{{x}^{1-\sigma }}{1-\sigma }$$

*Calculating utility*: To derive the utility of staying with and without implementing dry flood-proofing measures, we take the integral of the summed and time discounted utility under all possible events *i*. These events have a probability *p*_*i*_ of (no) flooding derived from Ward et al.^75^ Each household is assigned a position in the income distribution. Household income (*Inc*) is sampled from a lognormal distribution constructed using the income data of the department of residence and the position of the household in the income distribution^82^. We calculate the wealth (*W*) of each agent at time *t* using the income-to-wealth ratios per income quintile^83^.

*Bounded rationality*: Bounded rationality is captured by risk perception factor *β*. This perception factor results in both underestimations of flood hazard during periods of no flooding (*β* < 1) and overestimations of flood hazard immediately after a flood event (*β* > 1). We follow the DYNAMO setup by de Ruig et al.^35^ and Haer et al.^62^ and define risk perception as a function of the number of years after the most recent flood event, shown here in Eq. (5).

5 $${\beta }_{t}=c *{1.6}^{-d*t}+0.01$$

The function describes the evolution of risk perception factor *β* over *t* years after a flood event. We calibrate the maximum overestimation of risk *c* on a survey on the implementation of dry flood-proofing measures^48^. A description of this procedure is provided in Supplementary information S2.1**.** We simulate the changes in risk perception for each exposed household by modeling stochastic flood events in each coastal node based on return periods. To generate a flood, we iterate through each coastal node in each time step and sample a value between 0 and 1 from a uniform distribution. For example, if this value lies between 0.02 and 0.04, we simulate a flood for all households in this node residing in the 1/50-year flood zone. If this value exceeds 0.1, no flooding is simulated in this department when we assume a flood protection standard of 10 years. All households within an affected zone are then flooded.

*Flood damage*: We assume a coastal protection standard of 1/10 years for all coastal areas^45^ and exclude flooding with higher return periods from our analysis. We also assume the government keeps investing in flood protection under the different SLR scenarios, and the protection standard thus remains 1/10 years in the future. A protection standards of 5 and 25 years is tested in our sensitivity analysis. To derive flood damage associated with flood event *i* for each household, the household samples the inundation level of their current location during a 1 in 20, 50, 100, 200, 500, and 1,000-year flood from coastal flood maps produced in the AQUEDUCT flood analyzer framework^75^. We calculate an annual increase in inundation levels under SLR by interpolating between historic and projected inundation levels for 2080 under the RCP 4.5 and 8.5 climate change scenarios^74^. We calculate the expected damage *D* for each flood event *i* using the average maximum damage per building specific to France and depth-damage curves for residential buildings, both described by Huizing et al.^72^ Dry flood-proofing measures reduce flood damage by preventing floodwaters from entering a building. For this, we alter the depth-damage functions such that damage for inundation levels below 1 m is reduced by 85%^84^. Inundation above 1 m overtops the dry flood proofing, resulting in full damage.

*Cost of flood proofing*: In determining the costs of dry flood-proofing measures ${C}^{building},$ we use an average adaptation cost of EUR 10,800 euros per building based on Hudson^30^. This cost includes installing pumps and water barriers. Annual payments for the dry flood-proofing measures (${C}_{annual}^{building}$) are calculated using the formula presented in Eq. (6) and depend on the dry flood-proofing cost per building (${C}_{0}^{building}$), a fixed interest rate (*r*), and loan duration (*n*).

6 $${C}_{annual}^{building}={C}_{0}^{building}*\frac{r*{\left(1+r\right)}^{n}}{{\left(1+r\right)}^{n}-1}$$

*Budget constraints*: In defining the budget constraint for dry flood-proofing investments, we follow an expenditure cap definition of affordability by Hudson^85^ and assume households can invest a fixed percentage of their disposable income. Hudson^85^ distinguishes between investment affordability, entailing a household’s ability to pay for adaptation in a single upfront payment, and payment affordability, which applies when a series of annualized payments are made for a single measure. We apply a payment affordability definition and assume households obtain personal loans to finance dry flood proofing. If the annual loan payment exceeds the expenditure cap of the household, the household cannot afford to invest in dry flood proofing. We calibrate the loan duration, interest rate, and expenditure cap on the observed implementation rate of dry flood-proofing measures.

*Migration decisions*: Following Eq. (3), push factors (increasing coastal flood damage) and pull factors (income differentials *Inc*_*x,t*_ and amenities *A*_*x*_) interact with mooring factors (fixed migration costs) and shape the migration decisions of households in the coastal zone. These factors are calculated as follows. Each node *y* contains information on income distributions, amenity values, and a distance matrix to all other nodes. The amenity value of node *y* is a function of the distance to the coastline and wealth. We derive the monetary value of these coastal amenities from hedonic pricing studies based on the distance to the coast (see the section *Amenity value* below).

*Expected income and cost of migration*: For each node *y*, households sample the expected income *I*_*y*_ based on their current position in the lognormal income distribution constructed with department-level income data^82^. We assume this position in the income distribution is associated with profession; a household having an income in the lowest decile in its current department will not earn an income in the highest decile of the distribution in another department.

Migration costs *C*^*migration*^ to department *y* are a function of geographical distance and fixed migration costs (e.g., psychological costs when they leave friends and relatives and move to unfamiliar surroundings^53^). We capture this latter “place attachment cost” with a fixed monetary cost of migration *C*^*fixed*^. Ransom^86^ estimates this fixed cost to range from USD 105,095 to USD 140,023 for movers in the United States and estimates the total costs of a 500-mile move as between USD 394,446 and USD 459,270. Kennan and Walker^87^ estimate the fixed cost of migration at USD 312,146 for the average mover in the United States. Based on these figures, we construct a logit function and set the fixed cost of migration at EUR 250,000, which increases to a maximum migration cost of EUR 500,000 (Eq. 7). Due to the high uncertainty associated with migration costs, we test other values of migration costs in our sensitivity analysis.

7 $${C}_{y}^{migration}=\frac{2*{C}^{fixed}}{1+{e}^{-0.05*dis{t}_{xy}}}$$

*Amenity value*: We derive the amenity value of living near the coastline based on hedonic pricing studies of property values in coastal areas. Muriel et al.^88^ analyze transactions for coastal homes in the town of Finistère, on the Atlantic coast of France. They find that households are willing to pay a premium of 78% for a house with a good sea view compared to a house with no sea view and that a 1% increase in distance from the coastline results in a 0.087% decrease in property value. An increase of 10% in the distance from the coast would thus result in a decrease of 0.87% in property price premiums. In San Diego County (California, United States), Conroy and Milosch^89^ find a property price premium of 101.9% for houses within 500 feet (~ 150 m) of the coastline, 62.8% for houses between 500 and 1,000 feet (~ 150–300 m), and a decrease to 3.3% for property between 5 and 6 miles (~ 8–10 km). The researchers find no price premium for properties located further than 6 miles from the coast. Based on Conroy and Milosch^89^ and Muriel et al.^88^, we construct a distance decay function of coastal amenities (Supplementary information S2.2). Households residing within 500 m of the coastline experience a coastal amenity premium of 60% of their wealth, which decreases to 3% when located 10 km from the coast.

*Natural population change*: To account for natural population dynamics, we use net natural population change rates available for departments in 2013 and a medium population growth scenario^52,90^. A more detailed description of this procedure is provided in Supplementary information S1.3.

### Gravity model

Migration between inland nodes and toward coastal nodes is simulated using a gravity-based model of migration^21^. We expand the traditional gravity model of population (*Pop*) and distance (*Distance*) with income (*Inc*) and a coastal dummy variable (*Coastal*) to capture the effects of income differentials and coastal amenities on migration flows. The same drivers are also included in the ABM. The only differences are: (a) the use of flood risk information as a driver for migration and adaptation decisions in the ABM (because this only pertains to agents in flood zones), and (2) the use of population size in a department in the gravity model also influences migration decisions, whereas the size of population in the ABM only has an influence on the size of the flow. A table of the factors included in both models is provided in Table 1. A more detailed description of this procedure is provided in Supplementary information S2.

8 $$\begin{aligned} {\text{Ln}}\left( {Flow_{ij} } \right) & = \beta_{0} + \beta_{1} *\ln \left( {Pop_{i} } \right) + \beta_{2} *\ln \left( {Pop_{j} } \right) + \beta_{3} *\ln \left( {Inc_{i} } \right) + \beta_{4} *\ln \left( {Inc_{j} } \right) \\ & \;\; + \beta_{5} *Coastal_{i} + \beta_{6} *Coastal_{j} + \beta_{7} *\ln \left( {Distance_{ij} } \right) \\ \end{aligned}$$

**Table 1:** Push, pull and mooring factors in the gravity model of migration and ABM.

PPM factor	Included in ABM	Included in gravity model	Remarks
Population	No	Yes	ABM: Currently, population in the destination node is not a pull factor influencing migration decisions in the ABM. However, the migration flow out of the coastal node does emerge from the population residing in the node, hence, population has an effect on the size of the flow
			Gravity model: Population is included in the gravity model of migration
Household income	Yes	Yes	ABM: Current household income and expected income in other nodes in part drives migration decisions in the ABM
			Gravity model: Median household income in both origin and destination are included as factors in the gravity model
Coastal amenities	Yes	Yes	ABM: Households in the ABM experience coastal amenity value based on their current location. The agents also evaluate coastal amenity value in other departments when calculating the expected utility of migration
			Gravity model: Coastal amenities are captured in a coastal dummy variable for regions adjacent to the coastline
Migration costs	Yes	Yes	ABM: Agents evaluate a fixed migration cost and a migration cost that increases over distance in their decisions
			Gravity model: Distance between departments is included in the gravity model. This is considered a proxy for migration cost
Flood risk	Yes	No	ABM: A flood risk model that calculates expected flood damages for different return periods is integrated in the ABM
			Gravity model: Currently flood risk is not included in the gravity model of migration. The gravity model only simulates flows from outside of the flood zone. We assume that households living in inland areas do no consider flood risk in their migration decisions

### Coastal in-migration

Migration flows generated by the gravity model and the ABM are added to the receiving department at the beginning of each time step. Since we focus on household decisions under increasing coastal flood risk, people moving toward inland nodes are not required to be spatially explicit. Creating a spatially explicit agent population for all of France would increase the computational demand to an unfeasible level, so we aggregated households in the inland nodes. People moving toward the coastal nodes are grouped into households and made spatially explicit in the 1/100-year flood zone. Each household entering the coastal node from an inland node is assigned a position in the income distribution sampled from the closest 20 households residing within a 1 km radius. Households entering the coastal node from another coastal node maintain their current position in the income distribution.

Households are spatially allocated within the coastal flood zone based on the expected utility of 20 randomly selected cells. The agent assesses in each cell the current flood risk (mediated by their risk perception) and coastal amenity value. The agent is then allocated to cell with the highest subjective expected utility, consistent with decision framework applied throughout the ABM. A more detailed description of this procedure is provided in Supplementary information S1.4. Since urban spatial growth is not the focus of this paper, we limit population growth to raster cells classified as villages and cities, as in SMOD 2015^91^.

### Sensitivity analysis

We perform a one-at-a-time sensitivity analysis to assess the effects of uncertainties in fixed migration cost, flood protection standards, risk perceptions, and conversion of migration intentions to migration behavior. We use a spin-up period of 15 years to create an initial agent population of households that have partially implemented dry flood-proofing measures. Since the model contains several random processes, we generate 50 Monte Carlo runs with identical model settings for each parameter set. The mean value of these model runs is used for further analysis. To calculate the effect of SLR on coastal migration decisions, we compare the projected population residing in the 1/100-year flood zone under a baseline scenario of no SLR with EAD and population projections under RCP 4.5 and 8.5.

## Supplementary Information

Supplementary Information 1.

Supplementary Information 2.

## Data Availability

All input data used in this model can be obtained from the original data sources described in the methodology.

## Code availability

All model code is available from https://github.com/ltierolf/DYNAMO-M and https://doi.org/10.5281/zenodo.7656577.
