# Supplementary material for: A coupled agent-based model for France for simulating adaptation and migration decisions under future coastal flood risk
Source: Sci Rep. 2023 Mar 13;13:4176. doi: 10.1038/s41598-023-31351-y (PMC10011601; doi:10.1038/s41598-023-31351-y)
Supplement: Supplementary file 1 — Supplementary Information 1. [file 41598_2023_31351_MOESM1_ESM.pdf]

ODD+D protocol DYNAMO-M

This document describes the model, agents, and household decision-making in DYNAMO-M following the ODD+D protocol as described by Müller et al. (2013).

1. Overview

1.1 Purpose

1.1.a What is the purpose of the study?

The purpose of DYNAMO-M is to provide a novel modelling approach to simulate coastal adaptation and migration under sea level rise. By simulating both local adaptation and migration decisions under increasing flood risk we aim to provide a more comprehensive representation of household behavior than commonly applied risk models in studies on sea level rise. The model is applied to France.

1.1.b For whom is the model designed?

The model is designed for scientists and Disaster Risk Reduction (DRR) practitioners to increase their understanding of interactions between local household adaptation and migration under scenarios of future sea level rise.

1.2 Entities, state variables, and scales

1.2.a What kinds of entities are in the model?

DYNAMO-M is an agent-based model (ABM, see figure 1), which handles household migration and adaptation decisions in the coastal floodplain. In this version, the ABM is coupled to a gravity model, which simulates large regional flows of inland migration and migration towards the coastal floodplain. In this section we present the entities in both models.

ABM - HouseholdAgents

The core of DYNAMO-M is an agent-based model (ABM) that models decisions of spatially explicit households living in the 1/ 100-year coastal flood zone. Agents may undertake no action, implement damage reducing measures, or migrate to another *InlandNode* or *CoastalNode* (see also section: gravity model). *HouseholdsAgents* in the flood zone are generated using a gridded population map and an average household size specific to the case study area. Household attributes, such as, income

and wealth, are parameterized using local census data. In this model application to France, we simulate a total of 78,400 spatially explicit household agents in t = 0. The total number of agents varies over time because of natural population change and migration dynamics.

Gravity model – *CoastalNodes* and *InlandNodes*

A gravity model of migration between all 96 regional departments in France is used to account for coastward and inland migration. Each *InlandNode* represents the households in inland departments not bordering the sea, and the households residing in a department bordering the sea but *not* living in the flood plain. Each *CoastalNode* represents the aggregated households in the coastal floodplain within a coastal department. Thus, a coastal department both has an inland- and a coastal node, whereas inland departments only have inland nodes.

While the ABM simulates migration from the floodplain to *InlandNodes* or *CoastalNodes,* the gravity model simulates migration between *InlandNodes,* and towards *CoastalNodes*. The *CoastalNode* has an intermediary function to couple the gravity model to the ABM: households that migrate from an *InlandNode* towards a *CoastalNode* are added as 'flood zone agents' to the ABM, while households that migrate away from the coast (simulated by the ABM) do not need to flow through *CoastalNodes* but are instead directly absorbed in the chosen *InlandNodes*. Both the *InlandNodes* and the *CoastalNodes* thus hold the aggregated population information of their respective region. For the *InlandNodes*, this is based on already aggregated data, for the *CoastalNode* it is aggregated from the agent population.

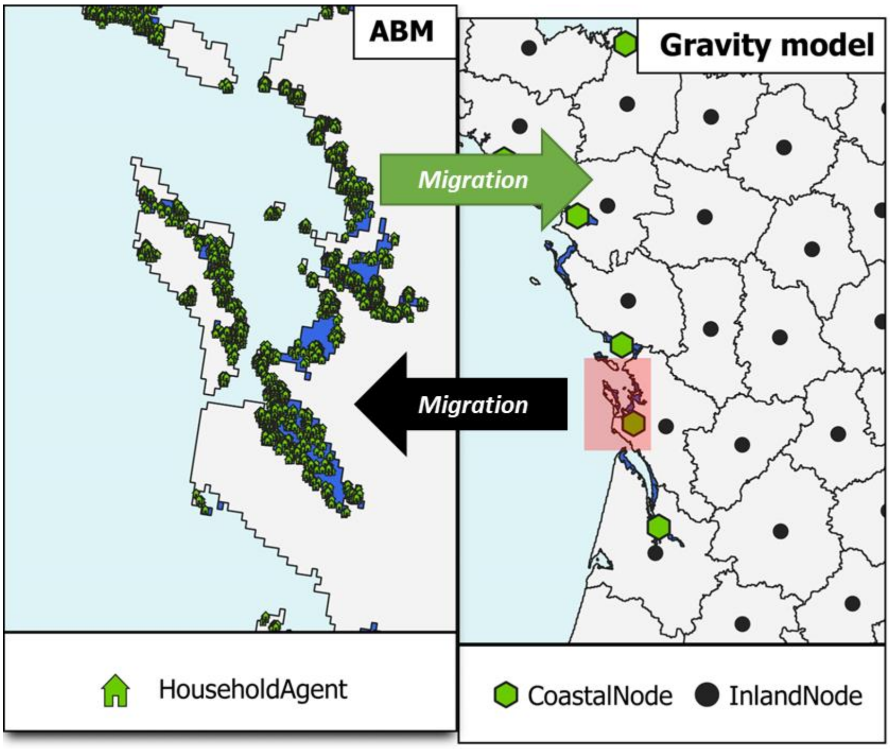

Figure 1 The two coupled models of DYNAMO-M. Household adaptation and migration (within the coastal floodplain -indicated in blue- and to inland departments) is simulated by the ABM. Inland migration between departments (black nodes) and migration towards the coastal floodplain (green nodes) is simulated by a gravity model. HouseholdAgents represent individual households residing in the coastal floodplain, whereas CoastalNodes and InlandNodes represent aggregated households of the subnational administrative unit (see section 1.2). This figure was generated using QGIS 3.22.13 (QGIS Association: https://qgis.org/).

1.2.b By what attributes (i.e. state variables and parameters) are these entities characterized?

Each *HouseholdAgent* is characterized by a geospatial location in the flood zone, which determines their interactions with the environment. A full description of all attributes of a *HouseholdAgent* is provided in table 1. The *HouseholdAgents* make decisions based on Subjective Expected Utility Theory (SEUT, see section 3.4 Submodels).

CoastalNodes and *InlandNodes* contain aggregated information, such population size and the number of households. They also contain an income distribution derived from local census data, a distance matrix of distance to all other nodes, and a dummy variable indicating whether the subnational administrative unit is adjacent to the coast. Migration flows between *InlandNodes* and towards *CoastalNodes* are modelled by the gravity model using utility maximization (see section 3.4 Submodels).

Table 1. Description of *HouseholdAgent* attributes

Parameter	Description
location	Georeferenced coordinates of household agent's location
household_size	Number of individuals within the household agent
income_percentile	Agent's position in the income distribution of the administrative unit (expressed in percentile)
income	Agent's disposable household income (in EUR)
wealth	Agent's wealth (in EUR)
property_value	Maximum flood damage to household property (fixed value, part of agent wealth)
amenity_value	The coastal amenity value experienced by the agent in their current location
water_levels	Dictionary containing the inundation levels for the agent's current location for all return periods
adapted	Binary variable describing whether the agent has implemented dry floodproofing measures
years_since_adaptation	Years passed since implementing the dry floodproofing measure. Used to determine whether the lifespan of the measure has been exceeded.
risk_perception	Agent's risk perception parameter converting objective flood probabilities to perceived probabilities
years_since_flood	State variable storing the years since the last flood experienced by the agent. Used to update the risk perception parameter
decision_horizon	Agent's decision horizon applied in the time discounting of expected utility
fixed_migration_cost	Monetized term capturing place attachment and physical costs of migration to another region.
r	Time discounting factor applied in time discounting of expected utility
risk_aversion	Agent's relative risk aversion constant.

1.2.c What are the exogenous factors / drivers of the model?

There are two exogenous drivers in the model: 1) sea level rise, captured by an increase of inundation levels associated with return periods ranging from 2- 1,000 years under different climate change scenarios (van Vuuren et al., 2011; Ward et al., 2020), and 2) aggregated population development as projected in the World Population Prospects (United Nations, 2019). For the latter, we only apply one medium population growth scenario. The spatial distribution of household agents is driven by migration processes in the model.

1.2.d If applicable, how is space included in the model?

Space is included as 1x1km2 gridded cells in which households are located and inundation levels are simulated.

1.2.e What are the temporal and spatial resolutions and extents of the model?

The model runs in subnational administrative units and can be implemented at a larger country or regional scale. It simulates household decisions in yearly timesteps spanning 65 years between 2015 and 2080. Inundation maps have a horizontal resolution of 1 square km and a vertical resolution in centimeters.

1.3 Process overview and scheduling

1.3.a What entity does what, and in what order?

ABM

In each timestep representing one year, all flood hazards maps are updated by interpolating the inundation depths for each raster cell between current and future climate conditions. Then, households that have decided to move are removed from the agent population. Next the ABM spatially distributes new household agents generated to meet population growth projections and distributes households that decided to move into the floodplain from *InlandNodes* (simulated by the gravity model) and from other *CoastalNodes* (simulated by the ABM) in the previous timestep. Households moving into the floodplain each calculate the subjective expected utility in a number of randomly selected urban cells within the floodplain based on their risk perception, objective flood risk in the cell, and amenity value. The agents are then allocated in the with the highest subjective expected utility. Then, using annual flood hazards maps, all household agents sample the inundation levels in their current location for flood events with return periods of 2 to 1,000 years. A submodel is then used to simulate stochastic flood events in each subnational administrative unit. These events are assumed to occur independent. This is done by a random draw from data on the different return periods of flood events (see section 3.4, submodels). Agents affected by flooding store the current year in memory, and all agents update their risk perception parameter based on the time passed since they last experienced a flood event. Next the ABM iterates through all *HouseholdAgents* to calculate the subjective time discounted expected utility of the three behavioral strategies (no action, adapt or migrate). This process is shown in more detail in figure 2.

Gravity model

The gravity model is applied to simulate migration flows between *InlandNodes* and towards *CoastalNodes*. First, households that have migrated in the previous timestep, or are deceased to meet population projections, are removed from each *InlandNode*. Then households moving into the *InlandNodes,* and households that are generated to meet population projections, are added to its aggregated household population. The model iterates through each *InlandNode* and calculates the migration flow to all other *InlandNodes* and *CoastalNodes*. The households moving towards *CoastalNodes* are added to the ABM in the next timestep. Households moving towards *InlandNodes* are added to the aggregated household population.

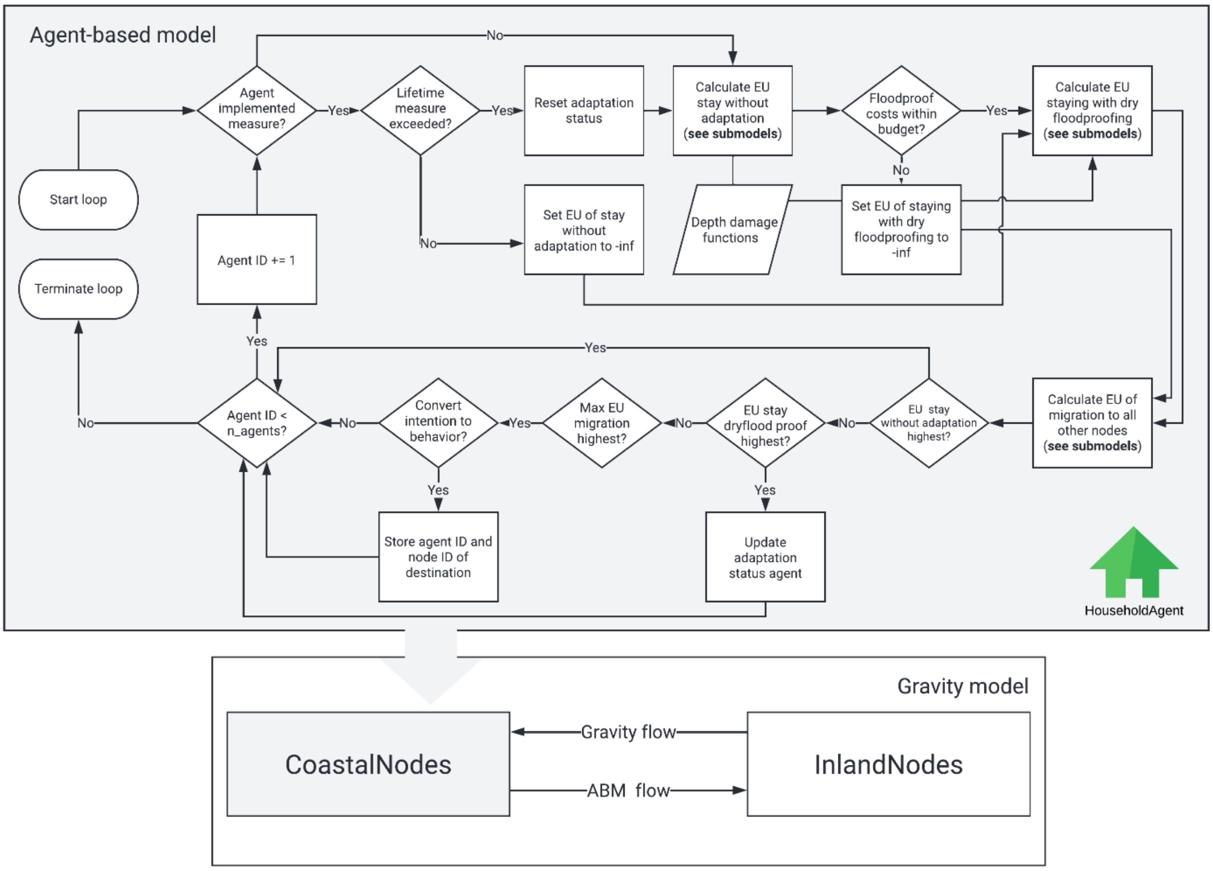

Figure 2. Scheduling process of individual *HouseholdAgents* in the ABM. The ABM loops through all agents in the coastal zone and first checks the adaptation status of the agent. Then, current flood risk is calculated and together with household risk perception fed into the utility calculations of staying with and without implementing floodproofing measures. Next the expected utility of migration to all other (coastal and inland) nodes is calculated. The agent executes the strategy that yields the highest subjective expected utility. The gravity model then projects migration between *InlandNodes* and towards *CoastalNodes.*

Design concepts

1.4 Theoretical and Empirical Backgrounds

2.1.a Which general concepts, theories or hypotheses are underlying the model's design at the system level or at the level(s) of the submodel(s)? What is the link to complexity and the purpose of the model?

The modelling approach presented in DYNAMO-M views migration as an alternative adaptation strategy as a response to sea level rise next to flood proofing buildings or 'no adaptation' (Black et al., 2011; Hauer et al., 2020). The choice of which adaptation strategy to apply is shaped not only by increasing flood hazard, but also by economic and political factors, and individual characteristics. In this study we aim to improve the representation of human behavior in flood risk assessment by including economic factors, such as, income differentials and amenity value, together with individual factors, such as, household perceptions of flood risk and risk aversion. We apply an agent-based model to deal with heterogeneity in adaptation behavior considering a dynamic risk perception and heterogeneity in household income and wealth.

2.1.b On what assumptions is/are the agents' decision model(s) based?

Household decisions in DYNAMO-M are grounded in a framework of subjective expected utility theory (Fishburn, 1981; Friedman & Savage, 1948; von Neumann & Morgenstern, 1953). Literature has shown that humans are bounded in their rationality, and act on the limited information available to them (Fishburn, 1981). In the context of flood adaptation, the perception of flood risk often determines whether people invest in flood adaptation measures (Aerts, 2020). Flood risk perception is dynamic over time, with people generally underestimating flood probability in absence of flooding and overestimating flood probability after just having experienced a flood event (Aerts et al., 2018). To account for this bounded rational behavior, we calculate a subjective expected utility by using a dynamic risk perception parameter that adjusts objective flood risk based on flood experience (de Ruig et al., 2022; Fishburn, 1981; Haer et al., 2020; Schrieks et al., 2021).

2.1.c Why is a/are certain decision model(s) chosen?

Subjective Expected utility theory (SEUT) is a well-established framework for simulating adaptation and migration decisions under uncertainty and risk in agent-based models of coupled human-natural systems (Bell et al., 2019; De Koning & Filatova, 2020; de Ruig et al., 2022). SEUT allows for a weighing of different adaptation strategies as a function of wealth and the costs and benefits under each strategy (Schrieks et al., 2021). This framework of SEUT was chosen as it allows for a direct weighing of the three behavioral strategies analyzed in this study, while accounting for bounded rational behavior.

2.1.d If the model / a submodel (e.g. the decision model) is based on empirical data, where does the data come from?

We calibrate the risk perception parameter on survey data from (Poussin et al., 2013). From this data, we use the implementation rate of various floodproofing measures for 885 households in France.

2.1.e At which level of aggregation were the data available?

The survey data of Poussin et al. (2013) is available at the household level.

1.5 Individual Decision Making

2.2.a What are the subjects and objects of decision-making? On which level of aggregation is decision-making modeled? Are multiple levels of decision making included?

In DYNAMO-M households make individual decisions to implement dry floodproofing or to migrate to any other node. Only a single level of decision making is included.

2.2.b What is the basic rationality behind agents' decision-making in the model? Do agents pursue an explicit objective or have other success criteria?

Household agents aim to maximize subjective time discounted utility, which is a function of wealth, coastal amenity, flood damages, and the costs of the different adaptation strategies. They reach this goal by either moving or implementing flood damage reducing measures.

2.2c How do agents make their decisions?

Agents in DYNAMO-M make decisions based on subjective expected utility theory. They weight the utility outcomes of no action, staying and implementing dry floodproofing, or migrating to all other nodes. Households choose the strategy with the highest utility outcome.

2.2.d Do the agents adapt their behavior to changing endogenous and exogenous state variables? And if yes, how?

The only exogenous variable driving household decisions are increasing flood damages under sea level rise. We assume households know the inundation levels in the current timestep and project these into the future to formulate predictions of future risks. Households thus adapt their behavior to changing coastal flood risk. Population development affects the number of household agents in residing in the coastal floodplain, resulting a potential (de-) in-crease of the exposed household population. A larger population, furthermore, increases the migration flow towards the flood zone, as population size has a positive effect on migration flows modeled by the gravity model of migration (see subsection 3.4).

2.2.e Do social norms or cultural values play a role in the decision-making process?

No.

2.2.f Do spatial aspects play a role in the decision process?

Households calculate flood risk based on inundation levels in their current location. Distance between nodes is a factor when calculating the migration costs between nodes.

2.2.g Do temporal aspects play a role in the decision process?

Households apply a decision horizon and a time discounting factor to calculate the subjective time discounted expected utility of each behavioral strategy. Inundation levels increase over time because of sea level rise, affecting the expected utility of decisions involving staying in their current location.

2.2.h To which extent and how is uncertainty included in the agents' decision rules?

We apply an intention to action parameter for agents deciding to migrate to another node (Chabé-Ferret et al., 2018). Using this factor, we randomly sample households that convert their migration intentions to migration action. The stochastic model for simulating random flooding event, account for uncertainty in future flood events.

1.6 Learning

2.3a Is individual learning included in the decision process? How do individuals change their decision rules over time as consequence of their experience?

Experience with flooding has an impact on the subjective expected utility of staying with and without implementing dry floodproofing measures. Experience with flooding results in a temporary overestimation of flood probabilities, whereas a lack thereof results in an underestimation of flood probabilities.

2.3.b Is collective learning implemented in the model?

No.

1.7 Individual Sensing

2.4.a What endogenous and exogenous state variables are individuals assumed to sense and consider in their decisions? Is the sensing process erroneous?

Households sense changes in coastal flood hazard and know their future income, wealth, and expected coastal amenities when migrating to other nodes based on their current position in the income distribution. Only the sensing of flood risk is erroneous, as it is affected by the risk perception parameter.

2.4.b What state variables of which other individuals can an individual perceive? Is the sensing process erroneous?

Households do not sense the state variables of other individuals.

2.4.c What is the spatial scale of sensing?

Households sense subjective flood risk and coastal amenity value in their current location. Households sense expected wealth, income, and coastal amenities for migration to all other nodes in the model. Flood risk in destination nodes is currently not sensed by the agent.

2.4.d Are the mechanisms by which agents obtain information modeled explicitly, or are individuals simply assumed to know these variables?

The mechanisms by which agents obtain information are modelled explicitly, sensing only happens locally.

2.4.e Are costs for cognition and costs for gathering information included in the model?

We include migration costs that monetizes the psychological costs of migration. This cost increases with distance between origin and destination node. Although we do not model the cost of gathering information explicitly, distance does constitute to a cognitive cost in evaluating the migration strategy.

1.8 Individual Prediction

2.5.a Which data uses the agent to predict future conditions?

Agents extrapolate the current conditions to make expectations of future conditions in each year within the time horizon.

2.5.b Might agents be erroneous in the prediction process, and how is it implemented?

Predictions of future flood risks are affected by the agent's current risk perception, resulting in under and overestimations of future flood probabilities. Projections of sea level rise are not included in the agents' predictions of future inundation levels.

1.9 Interaction

2.6.a Are interactions among agents and entities assumed as direct or indirect?

Interactions between agents and their environment (flood hazard) are mediated by their risk perception and adaptation status (whether they have implemented dry floodproofing measures). The number of individuals residing in a coastal node affects the number of households moving into the node via the gravity model, as the total population residing in a node is factor in the gravity model of migration.

2.6.b On what do the interactions depend?

Interactions depend on spatial location. Agents sample the inundation levels associated with flooding of different return periods based on their current location. Interactions between the environment are mediated by the agent's risk perception and adaptation status.

2.6.c If the interactions involve communication, how are such communications represented?

Interactions currently do not involve communication.

2.6.d If a coordination network exists, how does it affect the agent behaviour? Is the structure of the network imposed or emergent?

No coordination network exists.

1.10 Collectives

2.7.a Do the individuals form or belong to aggregations that affect, and are affected by, the individuals? Are these aggregations imposed by the modeller or do they emerge during the simulation?

Households residing outside of the coastal flood plain are aggregated in inland nodes. The cumulative household size (total population) affects migration flows projected under the gravity model of migration and affects the size of the migration flow emerging from household decisions in the ABM. Households residing in the 1/100-year flood zone are embedded in coastal nodes, each representing the coastal flood zone of a NUTS-3 region.

2.7.b How are collectives represented?

These collectives are represented as a different kind of entities in the gravity model (inland and coastal nodes). The collective shares state variables, such as, an income distribution and population size.

1.11 Heterogeneity

2.8.a Are the agents heterogeneous? If yes, which state variables and/or processes differ between the agents?

Household agents are heterogeneous in state variables (e.g., position in the income distribution, disposable income, wealth, experienced amenity value, risk perception) and their location in the flood zone (resulting in exposure to different flood depths). The decision to implement dry floodproofing measures is undertaken by individual households, resulting in a heterogenous vulnerability to flooding.

2.8.b Are the agents heterogeneous in their decision-making? If yes, which decision models or decision objects differ between the agents?

Decisions in the expected utility framework are a function of agent wealth, coastal amenities, cost of adaptation, and perceived flood risk. The decision to implement dry floodproofing is limited by a budget constraint. This means that the adaptation option is not accessible to low-income households.

1.12 Stochasticity

2.9.a What processes (including initialization) are modeled by assuming they are random or partly random?

During the initialization phase households residing in the floodplain are assigned a random position in the income distribution. In the spin up period used to initialize the agent population we run the model for 15 iterative timesteps whilst simulating random flood events based on flood probabilities. The spatial allocation of households in the coastal node is in part random to prevent all households moving towards the single cell with the highest expected utility. Households that are removed from the coastal node because of natural population decline are selected at random. The procedure for translating migration intentions to migration behavior selects a random subset of households from all households for which migration yields the highest utility outcome.

1.13 Observation

2.10.a What data are collected from the ABM for testing, understanding, and analyzing it, and how and when are they collected?

The total population of each node, the implementation rate of dry floodproofing measures for each coastal node, the total expected annual flood damages for each coastal node, and migration matrices from all nodes are tracked and exported after each timestep. Optionally all agents and their attributes, such as agent income, experienced amenity value, and location, can be exported each year.

2. Details

2.1 Implementation Details

3.1.a How has the model been implemented?

The model is implemented in Python 3.10.6.

3.1.b Is the model accessible and if so where?

Model code and documentation is made publicly available on Github, via https://doi.org/10.5281/zenodo.7057487

2.2 Initialization

3.2.a What is the initial state of the model world, i.e. at time t=0 of a simulation run?

In this section we describe to model application to France. Data sources may vary based on the case study location; however, the procedure remains roughly the same.

Inland and coastal nodes are constructed by overlaying polygon shapefiles of administrative areas with the 1/100-year flood zone of 2080 (GADM, 2022; Ward et al., 2020). For each node, households are sampled using gridded population data from the Global Human Settlement Layer of 2015 (GHSL) (Pesaresi & Freire, 2016). This procedure ensures that the total number of individuals within each node corresponds with the population in 2015. At t=0, a total of 18,233,835 households are aggregated in inland nodes and 77,391 households are embedded in the coastal nodes.

The gravity model is calibrated using migration matrices constructed using survey data on residential mobility (INSEE, 2017a). Income distributions for each node are constructed using income statistics on the department level and assuming a lognormal distribution (INSEE, 2016). A spin up period of 15 years is applied to initiate the adaptation status of agents in the coastal nodes. This spin up period is calibrated using survey data on the implementation rate of dry floodproofing measures in France (Poussin et al., 2013).

3.2.b Is initialization always the same, or is it allowed to vary among simulations?

During the spin-up period of the initialization phase flooding is simulated stochastically. This results in slightly different initial model conditions. When comparing scenarios, the random seed can be fixed, resulting in identical initial conditions.

3.2.c Are the initial values chosen arbitrarily or based on data?

Household income is based on local statistics data of French bureau of statistics (INSEE, 2016). Household wealth is determined based on the agent position in the income distribution following the factors described in Eurostat (2020). The amenity values are characterized based on the agent's distance to coast and hedonic pricing studies of Conroy & Milosch (2011) and Muriel et al. (2008). Fixed migration costs are based on (Kennan & Walker, 2011; Ransom, 2022). The lifespan of dry floodproofing set based on (Aerts & Botzen, 2011). The factor converting migration intention to migration behavior is based on findings from Lu (1999). Time discounting factors are based on Evans & Sezer (2005), a time horizon of 15 years is to reflect the average time a homeowner stays in his/her

home. The range of risk perceptions, loan duration, expenditure cap (budget constraint) and interest rates are chosen based on the benchmarking procedure on survey data described in the accompanying manuscript. Uncertainty in parameter values is addressed in the sensitivity analysis described in the accompanying manuscript.

2.3 Input Data

3.3.a Does the model use input from external sources such as data files or other models to represent processes that change over time?

The model makes use of externally modelled population change projections and changes in flood inundation depths under different climate change scenarios (United Nations, 2019; Ward et al., 2020). Other input data include gridded population maps to sample the agent population and regional census data to initialize household income.

2.4 Submodels

3.4.a What, in detail, are the submodels that represent the processes listed in 'Process overview and scheduling'?

<u>Submodel 1: Expected utility calculations:</u>

The agent executes the strategy yielding the highest subjective time discounted utility (DEU) within its budget constraints. The formulas for calculating the *DEU* of each strategy are as follows:

$$DEU_1 = \int_{p_i}^{p_I} \beta_t * p * U\left(\sum_{t=0}^{T} \frac{W_x + A_x + Inc_x - D_{x,t,i}}{(1+r)^t}\right) dp$$

$$DEU_2 = \int_{p_i}^{p_I} \beta_t * p_i * U\left(\sum_{t=0}^{T} \frac{W_x + A_x + Inc_x - D_{x,t,i}^{adapt} - C_t^{adapt}}{(1+r)^t}\right) dp$$

$$DEU_3 = U\left(\sum_{t=0}^{T} \frac{W_y + A_y + Inc_y - C_{y,t}^{migration}}{(1+r)^t}\right)$$

Utility is a function of household wealth (*W*), the amenity value of the current household location A_x, current household income I_x, expected damage *D* per event *i*, and adaptation costs C^{adapt}. Additional

variables for calculating the DEU of migration are prospected income *Inc* in destination node *y*, prospected amenity value in destination node A_y, and migration costs $C^{migration}$ to destination node *y*.

Bounded rationality is captured by risk perception factor *β*. This perception factor results in both underestimations of flood hazard during periods of no flooding (*β* < 1) and overestimations of flood hazard immediately after a flood event (*β* > 1). Risk perception as a function of the number of years after the most recent flood event, following the equation shown here:

$$\beta_t = c * 1.6^{-d*t} + 0.01$$

Expected wealth states are summed over time horizon *T* and discounted using discounting factor *r*. The household chooses to execute the strategy yielding the highest time discounted subjective utility *DEU*.

Submodel 2: Gravity mode of migration

Migration between inland nodes and toward coastal nodes is simulated using a gravity-based model of migration (Ramos, 2016). By including a model that simulates migration towards the floodplain, DYNAMO-M allows for studying the effects of changing push and pull factors on coastward migration under scenarios of sea-level rise. This provides a more realistic inflow of households towards the coastal floodplain than is achieved by only accounting for natural population growth. The theorical basis of gravity models of migration is generally represented as a random utility model (Khan et al., 2022; Ramos, 2016; Sheppard, 1978). The gravity model and the ABM are here embedded in a framework of utility maximization based on income differentials and coastal amenity values. Both models simulate migration decisions based on expected utility gains of migrating to another administrative unit.

A full description of this procedure is provided in the accompanying manuscript.

$$\ln(Flow_{ij}) = \beta_0 + \beta_1 * ln(Pop_i) + \beta_2 * ln(Pop_j) + \beta_3 * ln(Inc_i) + \beta_4 * ln(Inc_j) + \beta_5 * Coastal_i + \beta_6 * Coastal_j + \beta_7 * ln(Distance_{ij})$$

Submodel 3: Simulate flood event

To generate a flood, a value between 0 and 1 is sampled from a uniform distribution. If, for example, this value lies between 0.02 and 0.04, a flood is simulated for all households residing in the 1/50-year flood zone. If the value lies between 0.01-0.02, only the 1/100 zone households are flooded, etc. We assume a basic flood protection standard in all areas of 10 years. This means that a flood with a return period of 1/10 years does not cause any flooding. Thus, if the sampled value exceeds 0.1, no flooding is simulated in this department.

Submodel 4: Natural population change:

This procedure aims to match the total population in the model to the total population projected under the medium population growth scenario devised by the World Population Prospects 2019 (United Nations, 2019). The model does not simulate development of fertility rates into the future, but instead adjusts fertility rates of 2017 to match population development (INSEE 2017b). The natural population change rate *r* of each department *i* is adjusted with factor *a* to match the projected national population in the population growth scenario (see equation below). Adjustment factor *a* is optimized in each timestep by minimizing the squared residual between the modeled population change and the national population projections using a Nelder-Mead optimization algorithm (Gao & Han, 2010). Through this procedure, the relative differences in population change between departments are maintained.

s

$$r_i = \begin{cases} r_i * (1 + a) \text{ if } r_i \geq 0 \\ r_i * (1 - a) \text{ if } r_i < 0 \end{cases} \qquad \text{Eq. 1}$$

3.4.b What are the model parameters, their dimensions and reference values?

The parameter values can be found in table 2.

Table 2. Parameter values applied in DYNAMO-M application to France:

	Parameter	Description	Value	Reference
**ABM**	*B*	Risk perception	Ranges from 0 to 2	De Ruig et al. (2022)
	T	Decision horizon	15 years	De Ruig et al. (2022)
	W	Wealth	Varies between nodes and agents.	
	A	Amenity value	A function of distance to coast and agent wealth	Conroy & Milosch (2011); Muriel et al. (2008)
	Inc	Income	Varies between nodes and agents.	INSEE (2016)
	D	Damages	Property flood damages calculated using damage curves from	Huizinga et al. (2017)
	R	Time discounting factor	Set at 3.2%	Evans & Sezer (2005)
	C^{adapt}	Adaptation costs	€10,800, translated to annual costs with an interest rate of 4%	Aerts (2018)
	$C^{migration}$	Migration costs	Fixed €250,000 + distance related costs	Kennan & Walker (2011); Ransom (2022)
**Gravity model**	β_0	Intercept	-49.12	
	β_1	Weight of population in origin node	1.03	
	β_2	Weight of population in destination node	0.72	
	β_3	Weight of income in origin node	0.47	
	β_4	Weight of income in destination node	3.05	
	β_5	Weight of coastal dummy origin node	0.02	
	β_6	Weight of coastal dummy destination node	0.60	
	β_7	Effect of distance between origin and destination node	-1.02	

3.4.c How were submodels designed or chosen, and how were they parameterized and then tested?

The gravity model of migration is well established modeling framework of simulation migration flows (Anderson, 2011; Backhaus et al., 2015; Ramos, 2016). In this model application the gravity model was calibrated on migration matrixed derived from survey data and applied to simulate continued migration towards the coastal zone. Expected utility theory is a commonly applied decision theory in agent-based models of human environmental interactions and allows for incorporating bounded rational behavior by means of a risk perception parameter.

References

Aerts, J. C. J. H. (2018). A review of cost estimates for flood adaptation. In *Water (Switzerland)* (Vol. 10, Issue 11, p. 1646). MDPI AG. https://doi.org/10.3390/w10111646

Aerts, J. C. J. H. (2020). Integrating agent-based approaches with flood risk models: A review and perspective. *Water Security*, *11*(June), 100076. https://doi.org/10.1016/j.wasec.2020.100076

Aerts, J. C. J. H., & Botzen, W. J. (2011). Flood-resilient waterfront development in New York City: Bridging flood insurance, building codes, and flood zoning. *Annals of the New York Academy of Sciences*, *1227*(1), 1–82. https://doi.org/10.1111/j.1749-6632.2011.06074.x

Aerts, J. C. J. H., Botzen, W. J., Clarke, K. C., Cutter, S. L., Hall, J. W., Merz, B., Michel-Kerjan, E., Mysiak, J., Surminski, S., & Kunreuther, H. (2018). Integrating human behaviour dynamics into flood disaster risk assessment. *Nature Climate Change*, *8*(3), 193–199. https://doi.org/10.1038/s41558-018-0085-1

Anderson, J. E. (2011). The gravity model. *Annual Review of Economics*, *3*, 133–160. https://doi.org/10.1146/annurev-economics-111809-125114

Backhaus, A., Martinez-Zarzoso, I., & Muris, C. (2015). Do climate variations explain bilateral migration? A gravity model analysis. *IZA Journal of Migration*, *4*(1), 3. https://doi.org/10.1186/s40176-014-0026-3

Bell, A. R., Calvo-Hernandez, C., & Oppenheimer, M. (2019). Migration, Intensification, and Diversification as Adaptive Strategies. *Socio-Environmental Systems Modeling*. https://doi.org/10.18174/sesmo.2019a16102

Black, R., Bennett, S. R. G., Thomas, S. M., & Beddington, J. R. (2011). Migration as adaptation. *Nature*. https://doi.org/10.1038/478477a

Chabé-Ferret, B., Machado, J., & Wahba, J. (2018). Remigration intentions and migrants' behavior. *Regional Science and Urban Economics*, *68*, 56–72. https://doi.org/10.1016/j.regsciurbeco.2017.10.018

Conroy, S. J., & Milosch, J. L. (2011). An Estimation of the Coastal Premium for Residential Housing Prices in San Diego County. *Journal of Real Estate Finance and Economics*, *42*(2), 211–228. https://doi.org/10.1007/S11146-009-9195-X/TABLES/3

De Koning, K., & Filatova, T. (2020). Repetitive floods intensify outmigration and climate gentrification in coastal cities. *Environmental Research Letters*. https://doi.org/10.1088/1748-9326/ab6668

de Ruig, L. T., Haer, T., de Moel, H., Brody, S. M., Botzen, W. J. W., Czajkowski, J., & Aerts, J. C. J. H. (2022). How the U.S. can benefit from risk-based premiums combined with flood protection. *Nature Climate Change*.

Eurostat. (2020). Income, consumption and wealth—Experimental statistics (icw). In *European Statistical System (ESS)*. https://ec.europa.eu/eurostat/web/experimental-statistics/income-consumption-and-wealth https://ec.europa.eu/eurostat/cache/metadata/en/icw_esms.htm

Evans, D. J., & Sezer, H. (2005). Social discount rates for member countries of the European Union. *Journal of Economic Studies*, *32*(1), 47–59. https://doi.org/10.1108/01443580510574832

Fishburn, P. C. (1981). Subjective expected utility: A review of normative theories. *Theory and Decision 1981 13:2*, *13*(2), 139–199. https://doi.org/10.1007/BF00134215

Friedman, M., & Savage, L. J. (1948). The Utility Analysis of Choices Involving Risk. *Journal of Political Economy*, *56*(4), 279–304. https://doi.org/10.1086/256692

GADM. (2022). *GADM version 4.1*. https://gadm.org/download_world.html

Gao, F., & Han, L. (2010). Implementing the Nelder-Mead simplex algorithm with adaptive parameters. *Computational Optimization and Applications 2010 51:1*, *51*(1), 259–277. https://doi.org/10.1007/S10589-010-9329-3

Haer, T., Husby, T. G., Botzen, W. J. W., & Aerts, J. C. J. H. (2020). The safe development paradox: An agent-based model for flood risk under climate change in the European Union. *Global Environmental Change*, *60*, 102009. https://doi.org/10.1016/j.gloenvcha.2019.102009

Hauer, M. E., Fussell, E., Mueller, V., Burkett, M., Call, M., Abel, K., McLeman, R., & Wrathall, D. (2020). Sea-level rise and human migration. *Nature Reviews Earth & Environment*. https://doi.org/10.1038/s43017-019-0002-9

Huizinga, J., de Moel, H., & Szewczyk, W. (2017). Global flood depth-damage functions. Methodology and the database with guidelines. In *Joint Research Centre (JRC)*. Joint Research Centre (Seville site). https://ec.europa.eu/jrc%0Ahttp://publications.jrc.ec.europa.eu/repository/bitstream/JRC105688/global_flood_depth-damage_functions__10042017.pdf

INSEE. (2016). *Revenus et pauvreté des ménages en 2015*. https://www.insee.fr/fr/statistiques/3560121

INSEE. (2017a). *Migrations résidentielles: Localisation au département de résidence et au pays de résidence antérieure en 2017 − Logements, individus, activité, mobilités scolaires et professionnelles, migrations résidentielles en 2017 | Insee*. https://www.insee.fr/fr/statistiques/4508111?sommaire=4508161&q=residence

INSEE. (2017b). *Projections de population 2013-2050 pour les départements et les régions | Insee*. https://www.insee.fr/fr/statistiques/2859843

Kennan, J., & Walker, J. R. (2011). The Effect of Expected Income on Individual Migration Decisions. *Econometrica*, *79*(1), 211–251. https://doi.org/10.3982/ecta4657

Khan, M. A., Fatima, Z., & Fatima, S. (2022). Revisiting the Gravity Model of Migration. *Foreign Trade Review*, 00157325221088707. https://doi.org/10.1177/00157325221088707

Lu, M. (1999). Do People Move When They Say They Will? Inconsistencies in Individual Migration Behavior. *Population and Environment*, *20*(5), 467–488. https://doi.org/10.1023/A:1023365119874

Müller, B., Bohn, F., Dreßler, G., Groeneveld, J., Klassert, C., Martin, R., Schlüter, M., Schulze, J., Weise, H., & Schwarz, N. (2013). Describing human decisions in agent-based models—ODD+D, an extension of the ODD protocol. *Environmental Modelling and Software*. https://doi.org/10.1016/j.envsoft.2013.06.003

Muriel, T., Abdelhak, N., Gildas, A., & Francois, B. (2008). Assessing environmental benefits with the hedonic-price method: An application to coastal homes. *Economie et Prevision*, *185*(4), 47–62. https://doi.org/10.3406/ECOP.2008.7837

Pesaresi, M., & Freire, S. (2016). GHS-SMOD R2016A - GHS settlement grid, following the REGIO model 2014 in application to GHSL Landsat and CIESIN GPW v4-multitemporal (1975-1990-2000-2015). *European Commission, Joint Research Centre (JRC)*. https://data.jrc.ec.europa.eu/dataset/jrc-ghsl-ghs_smod_pop_globe_r2016a

Poussin, J. K., Botzen, W. J. J. W., & Aerts, J. C. J. H. (2013). Stimulating flood damage mitigation through insurance: An assessment of the french catnat system. *Environmental Hazards*, *12*(3–4), 258–277. https://doi.org/10.1080/17477891.2013.832650

Ramos, R. (2016). Gravity models: A tool for migration analysis. *IZA World of Labor*, *March*, 1–10. https://doi.org/10.15185/izawol.239

Ransom, T. (2022). Labor Market Frictions and Moving Costs of the Employed and Unemployed. *Journal of Human Resources*, *57*(S), S137–S166. https://doi.org/10.3368/JHR.MONOPSONY.0219-10013R2

Schrieks, T., Botzen, W. J. W., Wens, M., Haer, T., & Aerts, J. C. J. H. (2021). Integrating Behavioral Theories in Agent-Based Models for Agricultural Drought Risk Assessments. *Frontiers in Water*, *0*, 104. https://doi.org/10.3389/FRWA.2021.686329

Sheppard, E. S. (1978). Theoretical Underpinnings of the Gravity Hypothesis. *Geographical Analysis*, *10*(4), 386–402. https://doi.org/10.1111/j.1538-4632.1978.tb00666.x

United Nations. (2019). *World Population Prospects—Population Division—United Nations*. https://population.un.org/wpp/Download/Archive/Standard/

van Vuuren, D. P., Edmonds, J., Kainuma, M., Riahi, K., Thomson, A., Hibbard, K., Hurtt, G. C., Kram, T., Krey, V., Lamarque, J. F., Masui, T., Meinshausen, M., Nakicenovic, N., Smith, S. J., & Rose, S. K. (2011). The representative concentration pathways: An overview. *Climatic Change*, *109*(1), 5–31. https://doi.org/10.1007/S10584-011-0148-Z/TABLES/4

von Neumann, J., & Morgenstern, O. (1953). Theory of games and economic behavior. In *Theory of Games and Economic Behavior*. Princeton University Press. https://doi.org/10.2307/2981222

Ward, P. J., Winsemius, H. C., Kuzma, S., Bierkens, M. F. P. P., Bouwman, A., Moel, H. D., Loaiza, A. D., Eilander, D., Englhardt, J., Gilles, E., Gebremedhin, E. T., Iceland, C., Kooi, H., Ligtvoet, W., Muis, S., Scussolini, P., Sutanudjaja, E. H., Beek, R. V., Bemmel, B. V., … Luo, T. (2020). Aqueduct Floods Methodology. In *World Resources Institute* (January; pp. 1–28). https://www.wri.org/research/aqueduct-floods-methodology www.wri.org/publication/aqueduct-floods-methodology
