# Supplementary material for: A coupled agent-based model for France for simulating adaptation and migration decisions under future coastal flood risk
Source: Sci Rep. 2023 Mar 13;13:4176. doi: 10.1038/s41598-023-31351-y (PMC10011601; doi:10.1038/s41598-023-31351-y)
Supplement: Supplementary file 2 — Supplementary Information 2. [file 41598_2023_31351_MOESM2_ESM.pdf]

Supplementary information for:

A coupled agent-based model for France for simulating adaptation and migration decisions under future coastal flood risk

Lars Tierolf*, Toon Haer, W. J. Wouter Botzen, Jens A. de Bruijn, Marijn J. Ton, Lena Reimann, Jeroen C.J.H. Aerts

* Corresponding author: Lars Tierolf, Institute for Environmental Studies (IVM), Vrije Universiteit Amsterdam (VU), Boelelaan 1111, 1081 HV Amsterdam, The Netherlands; lars.tierolf@vu.nl

Contents

S1. Supplementary methods 2

S1.1 Calibration of the gravity model 2

S1.2 Coastal amenity function.. 5

S1.3 Population growth projections 5

S1.4 Spatial allocation of households in flood zone 6

S2. Supplementary results 7

S2.1 Calibration of the ABM.. 7

S2.2 Results for individual coastal nodes.. 10

S2.3 Migration and flood risk projections for two selected departments.............. 11

S2.3.1 Nord.. 12

S2.3.2 Charente-Maritime.. 12

S2.4 Sensitivity analysis 14

References 17

S1. Supplementary methods

S1.1 Calibration of the gravity model

We calibrate the gravity model using a survey based matrix of migration flows between departments (NUTS-3 regions) in France[1]. We follow the estimation approach of Benveniste et al.[2] and perform an ordinary least-squares (OLS) regression on the migration flows on the department levels and the explanatory variables population, household income, coastal adjacency and distance (shown in table S1). We select these variables based on a framework of utility maximization with variables reflecting income (*income*), coastal amenities (*coastal adjacency*) and migration costs (*distance*). We use income and population data from the national bureau of statistics[3,4]. Distance is computed as the distance between the centroid of each department[5]. We determine whether a department is adjacent to the coast using a coastline shapefile of the European Environment Agency (EEA)[6]. The regression yields an R-squared of 0.714 (adjusted R-squared of 0.713), shown here in table S2. All variables except coastal adjacency of the origin department are significant under the 0.05 significance level.

To assess whether the migration flows simulated by the ABM are consistent with migration flows produced by gravity model we provide a direct comparison of outmigration from coastal nodes as simulated by both models. We run the model two times for a period of 10 years. First, we apply the ABM to simulate household decisions under the Full model setting, considering a scenario of no SLR. Then, we run the model treating all nodes (coastal and inland) as inland nodes. The resulting flows out of each coastal node under both approaches are shown in Table S3.

Table S1. Variables included in the gravity model of migration

Variable	Description	Source
flow	Migration flow between departments	INSEE[1]
population_i, population_j	Population in each department	INSEE[4]
income_i, income_j	Median household income in each department	INSEE[3]
coastal_i, coastal_j	Binary indicating whether a department is adjacent to the coast	GADM[5] and EEA[6]
distance_ij	Distance between department centroids	GADM[5]

Table S2. Result from the OLS regression on department-level migration flows. Standard errors clustered around the mean are indicated within brackets.

	Effect on migration flows
Intercept	-49.12*** (1.854)
population_i	1.0286*** (0.014)
population_j	0.7201*** (0.014)
income_i	0.4780*** (0.140)
income_j	3.0502*** (0.142)
coastal_i	0.0250 (0.022)
coastal_j	0.5975*** (0.022)
distance_ij	-1.0245*** (0.015)
R-squared	0.714
Adj. R-squared	0.713
N. Observations	7992
Origin fixed effect	No
Destination fixed effect	No
Model	OLS
Method	Least Squares
**p < 0.05, **p < 0.01, ***p < 0.001*	

Table S3. One-on-one comparison of migration flows across models

Flood zone	Flow gravity model	Flow ABM
Pyrénées-Atlantiques	2	19
Charente-Maritime	145	192
Gironde	144	174
Landes	0	5
Pyrénées-Orientales	0	6
Aude	0	3
Gard	10	23
Hérault	66	88
Loire-Atlantique	42	43
Vendée	129	162
Bouches-du-Rhône	29	47
Var	2	7
Côtes-d'Armor	13	32
Finistère	28	63
Ille-et-Vilaine	113	182
Morbihan	2	8
Haute-Corse	1	22
Nord	367	495
Pas-de-Calais	220	343
Somme	27	62
Calvados	98	129
Eure	6	10
Manche	116	142
Seine-Maritime	340	369

S1.2 Coastal amenity function

Based on Conroy and Milosch[8], we construct a stylized function to calculate the monetary value of coastal amenities in the flood zone. Consistent with hedonic pricing studies, we assume this amenity to be a function of distance to the coast and household wealth. The interpolated function is plotted in Fig. S1. Households sample the amenity value at their current location and consider the amenity value experienced by household with the same position in the income distribution in other regions in the utility calculations of migration.

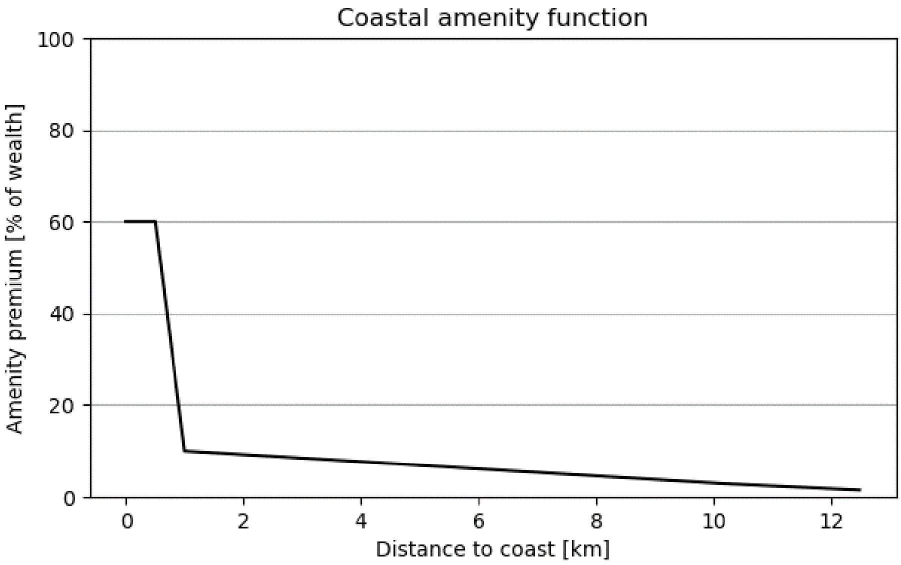

Figure S1 Coastal amenity function applied to determine the amenity value each agent experiences within the floodplain, and the amenity value used in calculating the expected utility of migration towards the floodplains. The amenity value is considered a function of agent wealth and distance to the coast

S1.3 Population growth projections

In this procedure, we match the total population in the model to the population projected under the medium population growth scenario devised by the World Population Prospects 2019[9]. Since we do not know how fertility rates will develop in the future, we adjust the natural population change r of each department i with factor a to match the projected national population in the population growth scenario (see equation below). In each time step, we optimize adjustment factor a by minimizing the squared residual between our modeled population change and the national population projections using a Nelder-Mead optimization algorithm[10]. Through this procedure, we maintain the relative differences in population change between departments.

$$r_i = \begin{cases} r_i * (1 + a) \text{ if } r_i \geq 0 \\ r_i * (1 - a) \text{ if } r_i < 0 \end{cases}$$

S1.4 Spatial allocation of households in flood zone

We allocate households spatially based on flood risk, coastal amenity, and risk perception to be consistent with the utility framework applied throughout the ABM. Each households assesses the subjective expected utility (*SEU*) in 20 randomly selected urban[11] raster cells by applying the following equation for each agents being allocated in the flood zone:

$$SEU_{cell} = U(A_{cell} - \beta * EAD)$$

Coastal amenity (*A*) is calculated by the function specified in S1.2. To account for current flood risk in the cell (expected annual damages, *EAD*), we create annual maps of expected damages by interpolating water levels of inundation of all return periods (accounting for the flood protection standard) between 2015 and 2080[12] . Then, for each year, we integrate the expected damage factor for residential buildings (based on Huizinga[13]) in each inundated cell over the return periods included in the analysis. The resulting expected annual damage factors are multiplied with the property value of the agent being allocated in the coastal flood zone to calculate the EAD. EAD in the cell is multiplied with the risk perception parameter of the agent moving in (*β*, see *Coastal flood zone decisions (ABM): Bounded rationality* in manuscript). The agent is then allocated in the cell with the *SEU*. We also tested this procedure with 15 and 20 random cells to assure increasing the number of cells did not affect aggregated model results.

S2. Supplementary results

S2.1 Calibration of the ABM

Following de methods by De Ruig et al.[14], we calibrate DYNAMO-M using survey data on the implementation of property-level dry flood-proofing measures using survey data from Poussin et al.[15] In the latter study, respondents in coastal and river floodplains in France were surveyed regarding the presence of different flood-proofing measures in their current home. Consistent with the Federal Emergency Management Agency (FEMA) definition of dry flood-proofing measures[16], we consider households to have implemented dry flood-proofing measures if they had installed any of the following measures:

1) The foundations of the house/building were strengthened against pressures due to floodwater;
2) The walls and equipment of the ground floor, such as the doors, insulation, and woodwork, were made with water-resistant materials;
3) Anti-backflow valves were installed on pipes to prevent water from entering the home;
4) A pump was installed to pump away water entering the home.

Poussin et al.[15] found that 36.05% of all respondents in the West, Var, and Ardennes regions had implemented any of the dry flood-proofing measures (Table S4). Since this percentage is determined by our definition of dry flood proofing, we also test alternative definitions. For example, only 9.96% of all respondents indicated they had a pump installed in their home. We, therefore, calibrate the adaptation rate (the percentage of households who implement adaptation decisions) to a range between 9.63% and 36.05%.

We calibrate the variables risk perception (β), expenditure cap, and loan interest rates (see Methods) to match the simulated adaptation rate to the observed adaptation rate. For this, we explore the parameter space of parameter ranges identified in the literature (Table S5) and assess the implementation rate of dry flood-proofing measures after forcing the model with a 1/100-year coastal flood, similar to the coastal flooding observed in 2010 during storm Xynthia[17]. Since the model contains several random processes (see Methods), the modeled dry-proofing implementation rate in three duplicate runs for each parameter setting is averaged. We used 2,970 unique model runs in the calibration procedure, for which the results are shown in Fig. S2. This figure shows density plots of

simulated adaptation rate for a selection of parameters tested in the calibration procedure. For example, as shown in the upper left panel, a risk perception of 2 results in a simulated adaptation rate of 9.63% to 36.05% under all parameter combinations of interest rates, loan durations, and expenditure caps assessed in the procedure. Multiple parameter combinations within the ranges identified in the literature (Table S2) resulted in a simulated implementation rate between 9.63% and 36.05%. Table S6 presents the benchmarked parameter settings used in the model.

Table S4. Observed implementation of dry flood proofing measure in the Var, Ardennes, and West regions (source: Poussin et al.[15])

Measures implemented	Percentage	Total number of respondents
Any measure	36.05	885
Foundations strengthened	20.46	694
Walls and ground flood equipment resistant to water	21.87	750
Anti-backflow valves installed	10.4	783
Pump installed	9.63	758

Table S5. Parameter ranges used in the calibration of household adaptation behavior

Parameter	Values tested	Sources
Peak risk perception	0 to 10 in steps of 1	de Ruig[14]
Loan interest rate	1 to 5% in steps of 1%	de Ruig[14]
Expenditure cap	2 to 10% in steps of 1%	Kousky and Kunreuther[18] find an expenditure cap of 5% of average income in the United States. We in addition test values between 2 and 10%.
Loan duration	10 to 30 years in steps of 2 years	We base the loan duration on the average mortgage length in the Eurozone (20 to 30 years) and include a loan duration of 10 years in our analysis.

Table S6. Calibrated parameter settings (see Equations in methods)

Parameter	Value
Peak risk perception	2
Loan duration	16 years
Loan interest rate	4%
Expenditure cap	6%

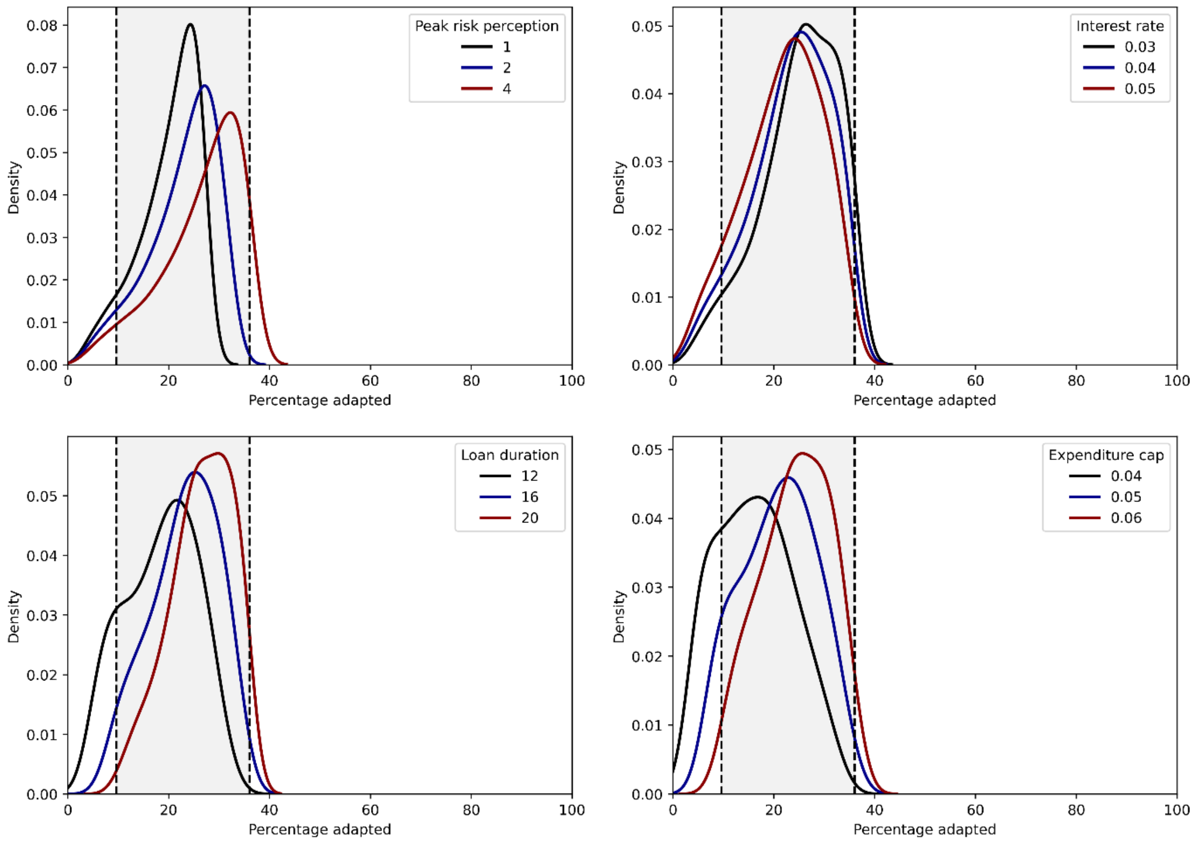

Figure S2. Density plots of all modeled observations showing the simulated implementation rate of dry flood-proofing measures. Modeled observations under a risk perception of 0 are omitted from this figure, as the implementation rate of dry flood-proofing measures was 0 under all model settings.

S2.2 Results for individual coastal nodes

Table S7. Projected population, EAD, and SLR induced emigrants in the 1/100-year floodplain. We show the median of 50 Monte Carlo model runs under the *Full* model setting.

Department	Population residing in the 1/100-year floodplain				EAD (million €)				SLR induced migrants	
	2015	*2080 (No SLR)*	*2080 (RCP4.5)*	*2080 (RCP8.5)*	*2015*	*2080 (No SLR)*	*2080 (RCP4.5)*	*2080 (RCP8.5)*	*2080 (RCP4.5)*	*2080 (RCP8.5)*
Nord	45,586	67,433	63,742	63,038	12	13	87	104	3,691	4,395
Seine-Maritime	39,444	73,365	72,439	72,121	20	28	54	61	926	1,244
Gironde	19,627	30,768	30,160	29,945	5	13	35	37	608	823
Manche	14,475	16,816	16,354	16,139	22	40	50	52	462	677
Pas-de-Calais	25,341	34,279	33,856	33,660	14	17	28	31	423	619
Ille-et-Vilaine	15,194	25,533	25,141	24,795	18	37	46	48	392	738
Calvados	11,967	19,721	19,358	19,334	16	35	50	55	363	387
Vendée	18,077	22,782	22,444	22,154	9	24	35	38	338	628
Charente-Maritime	20,624	22,607	22,335	22,321	10	9	18	20	272	286
Loire-Atlantique	7,049	11,678	11,495	11,447	4	10	20	22	183	231
Finistère	6,907	7,189	7,042	7,038	5	8	13	14	147	151
Hérault	10,751	14,917	14,781	14,798	5	12	15	15	136	119
Gard	2,657	2,727	2,624	2,612	4	5	16	19	103	115
Bouches-du-Rhône	5,402	6,842	6,769	6,774	1	1	3	3	73	68
Eure	1,233	2,205	2,137	2,111	1	3	4	5	68	94
Somme	4,119	6,733	6,693	6,613	1	1	7	8	40	120
Haute-Corse	2,114	1,533	1,512	1,507	6	9	10	11	21	26
Pyrénées-Atlantiques	1,754	1,386	1,365	1,360	2	1	2	2	21	26
Côtes-d'Armor	3,549	3,164	3,146	3,133	1	1	1	2	18	31
Morbihan	934	670	660	659	1	1	1	1	10	11
Var	1,013	782	775	775	0	0	0	0	7	7
Aude	266	193	189	187	0	0	0	0	4	6
Pyrénées-Orientales	569	334	331	326	0	0	1	1	3	8
Landes	528	297	295	288	0	0	0	0	2	9
Corse-du-Sud	79	79	79	79	0	0	0	0	0	0

S2.3 Migration and flood risk projections for two selected departments

To better understand the processes driving adaptation and migration decisions, we focus on adaptation and migration in two coastal departments: Nord and Charente-Maritime. We show individual model runs created under RCP 4.5 and analyze model projections of the population and EAD. The overall increase in the coastal population and EAD over time is visualized in the middle panels of Fig. S4. The experience of flooding drives migration and adaptation decisions through increased risk perceptions, illustrated here by a small dip in the population and a larger decrease in the EAD immediately after a flood event. The EAD decreases after each flood event, as overestimations of flood risk prompt households to migrate or implement dry flood-proofing measures. However, this reduction in the EAD is rapidly offset by an increase in the exposed population. A drop in the number of households having implemented dry flood-proofing measures is shown around 2075 when the 75-year lifespan of measures implemented during the 15-year spin-up period is exceeded.

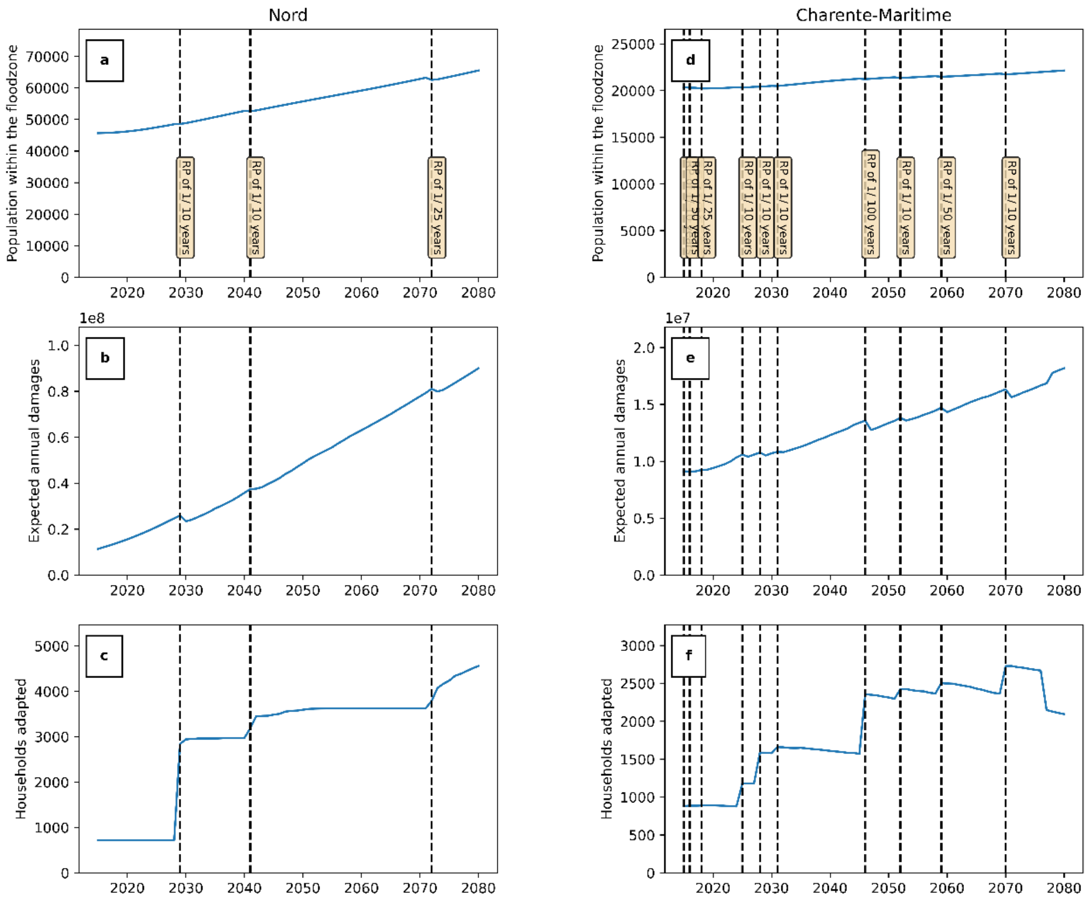

Figure S3. Projected population change and EAD for the 1/100-year flood zones of Nord (left panels: a, b, c) and Charente-Maritime (panels to the right: d, e, f) under RCP 4.5. Dashed vertical lines indicate stochastic flood events; labels show the exceedance probability of the event. The drop in the number of households having implemented adaptation measures in 2075 can be attributed to exceedance of the lifespan of adaptation measures implemented during the spin-up period.

S2.3.1 Nord

Nord is the most populated department of France, and we project most SLR-induced migration to occur here (3,663 migrants under RCP 8.5, Table S8). In this model run, the population residing in the 1/100-year flood zone of Nord increases from 45,676 people in 2015 to 65,503 people in 2080 under RCP 4.5 (Fig. S3a). The EAD increases from EUR 12 million to EUR 90 million in 2080 (Fig. S3b). The implementation of dry flood-proofing measures increases from 717 (5.23%) in 2015 to 4,556 households (21.36%) in 2080 (Fig. S3c). Most households migrate towards Paris and Hauts-de-Seine (Fig. S4, in green). This preference can be explained by a lower household income in Nord compared to the household income in the se destination departments.

S2.3.2 Charente-Maritime

Charente-Maritime is located on the Atlantic coast (Fig. S5, in red). The population residing in the coastal 1/100-year flood zone of Charente-Maritime increases from 20,360 people in 2015 to 22,148 people in 2080. The EAD increases from EUR 10 million in 2015 to EUR 18 million in 2080. The number of households having implemented dry flood-proofing measures increases from 881 (13.75%) in 2015 to 2,094 (25.25%) in 2080. Since the net fertility rate in this department is negative (−0.38% annually), only coastward migration increases the population exposed to coastal flooding. This negative fertility rate reduces the number of households that have implemented dry flood-proofing measures over time, as households are removed from the agent population and new households entering the floodplain have not implemented any measures. Households mainly migrate toward Paris and Hauts-de-Seine, but also to the floodplain of Loire-Atlantic (Fig. S4, in red), indicating that migration in this region is driven by income differentials and coastal amenities.

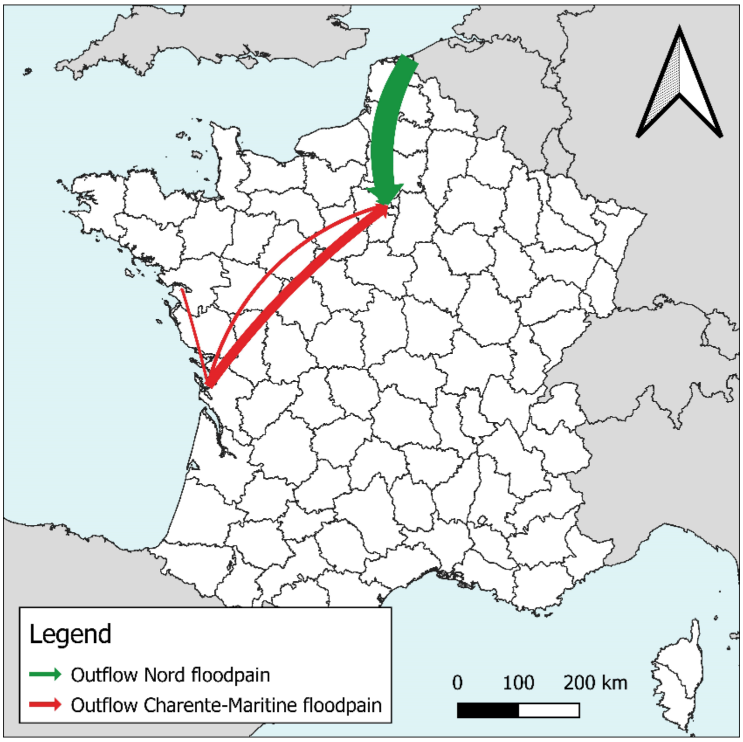

Figure S4. Migration flows from the floodplains of Nord and Charente-Maritime based on the mean of 50 Monte Carlo model runs. The width of the arrow indicates the relative size of the migration flow. This figure was generated using QGIS 3.22.13 (QGIS Association: https://qgis.org/).

S2.4 Sensitivity analysis

A one-at-a-time sensitivity analysis is performed to assess the model robustness to uncertainties in migration costs, flood protection standards, and conversion of migration intention to migration behavior on model projections. We run the model for two values of each parameter and assess the projections of the SLR-induced migration and EAD in 2080. Table S8 displays the mean of 50 Monte Carlo model runs.

Table S8. Results of the sensitivity analysis based on the mean of 50 Monte-Carlo model runs

Settings	Population residing in the coastal 1/100-year flood zone in 2080			EAD in 2080 (million €)			Number of SLR induced coastal migrants 2080	
	No SLR	*RCP4.5*	*RCP 8.5*	*No SLR*	*RCP 4.5*	*RCP 8.5*	*RCP 4.5*	*RCP 8.5*
Full model setting	374,033	365,721	363,215	267	497	549	8,345	10,934
Fixed migration cost of €125,000	245,058	242,354	241,220	199	373	414	2,704	3,837
Fixed migration cost of €500,000	525,852	523,492	522,825	370	684	759	2,361	3,028
1/5-year FPS	354,552	335,746	331,390	480	925	1,032	18,807	23,163
1/25-year-year FPS	387,400	384,979	384,015	148	275	275	2,420	3,385
Migration intention to behavior 6%	381,579	375,996	375,747	278	514	573	5,583	5,833
Migration intention to behavior of 24%	368,884	355,785	352,052	257	475	524	13,099	16,832
Max risk perception of 1	383,598	379,896	378,276	286	526	582	4,685	6,337
Max risk perception of 4	365,228	353,945	349,092	251	471	517	11,283	16,136

Fixed migration costs: Decreasing the fixed direct monetary and indirect psychological migration cost from €250,000 to €125,000 counterintuitively decreases the number of SLR-induced migrants under both RCP scenarios. This can be explained by the other drivers of migration that play a role in migration decisions in the ABM. Decreasing the cost of migration not only increases migration driven by flood risk, but also migration driven by income differentials and coastal amenity values. A lower place attachment results in more households choosing to migrate out of the floodplain towards areas with a higher median household income. This in turn lowers the exposed population to flooding, resulting in less SLR driven migration.

Increasing the fixed migration costs to €500,000 resulted in less SLR-induced migration compared to the baseline setting. The higher place attachment represented by this higher fixed migration cost resulted in less household migration away from the floodplain, increasing the population residing in the coastal floodplain compared to the *full* model settings. This increase in exposed households because of more population growth resulted a greater increase of coastal flood risk under all climate scenarios.

Flood protection standard: The flood protection standard (FPS) of coastal areas affected by SLR strongly affects migration decisions under SLR. Lowering the governmental maintained FPS from 1/10 to 1/5 years more than doubled the number of SLR- induced migrants under RCP4.5 and RCP8.5, whereas increasing the FPS to 1/25-year events resulted less coastal migration (a decrease of 69% under RCP 8.5 compared to the *full* model settings: from 10,934 to 3,385 migrants in 2080). However, the potential levee effect of this strategy should be considered in future research, as the coastal population exposed to flooding will continue to increase under SLR. This effect refers to the feature that governmental flood protection can reduce the incentive for autonomous adaptation by local households. Because of these investments, people in the flood zone feel safer, and are less inclined to migrate[19].

Intention to behavior: Our model is sensitive to changes in the factor translating migration intentions to migration behavior. Reducing the factor resulted in less SLR driven migration, whereas increasing the factor resulted in more SLR driven migration. This result highlights the need for a better characterization of migration intention to migration behavior in studies on SLR induced migration. Longitudinal surveys on the effect of migration intentions on actual migration behavior after the experience of a flood event will benefit the validation of these simulated dynamics[20].

Maximum flood risk perception: Setting the risk perceptions of households that just experienced a flood event to 1 (no overestimations of flood risk) resulted in less SLR driven migration and a higher EAD compared to the *full* model settings. This result indicates that even without overestimations of

flood risk, SLR could increase migration out of the coastal zone. When doubling risk perception to 4, the number of households choosing to migrate under SLR increases in both scenarios. Both results highlight a model sensitivity to risk perceptions, indicating that perceptions of risk should be accounted for in studies of SLR induced migration. Based on our calibration procedure described in S2.1, we choose a risk perception of 2, which results in an adaptation uptake rate that matches uptake rates found in the survey.

References

1. INSEE. Migrations résidentielles : localisation au département de résidence et au pays de résidence antérieure en 2017 − Logements, individus, activité, mobilités scolaires et professionnelles, migrations résidentielles en 2017 {\textbar} Insee.
2. Benveniste, H., Oppenheimer, M. & Fleurbaey, M. Effect of border policy on exposure and vulnerability to climate change. *Proc. Natl. Acad. Sci. U. S. A.* **117**, 26692–26702 (2020).
3. INSEE. Structure et distribution des revenus, inégalité des niveaux de vie en 2014 | Insee. https://www.insee.fr/fr/statistiques/3560118 (2017).
4. INSEE. Téléchargement du fichier d'ensemble des populations légales en 2017 − Populations légales 2017 | Insee. *Institut national de la statistique et des études économiques* 1295 https://www.insee.fr/fr/statistiques/4265429?sommaire=4265511 (2021).
5. GADM. Download GADM data (version 4.1). https://gadm.org/download_country.html (2022).
6. European Environment Agency. Europe coastline shapefile — European Environment Agency. https://www.eea.europa.eu/data-and-maps/data/eea-coastline-for-analysis-1/gis-data/europe-coastline-shapefile (2021).
7. Benveniste, H., Oppenheimer, M. & Fleurbaey, M. Climate change increases resource-constrained international immobility. *Nat. Clim. Change* **12**, 634–641 (2022).
8. Conroy, S. J. & Milosch, J. L. An Estimation of the Coastal Premium for Residential Housing Prices in San Diego County. *J. Real Estate Finance Econ.* **42**, 211–228 (2011).
9. United Nations. World Population Prospects - Population Division - United Nations. https://population.un.org/wpp/Download/Archive/Standard/ (2019).
10. Gao, F. & Han, L. Implementing the Nelder-Mead simplex algorithm with adaptive parameters. *Comput. Optim. Appl. 2010 511* **51**, 259–277 (2010).
11. Pesaresi, M. & Freire, S. GHS-SMOD R2016A - GHS settlement grid, following the REGIO model 2014 in application to GHSL Landsat and CIESIN GPW v4-multitemporal (1975-1990-2000-2015). *Eur. Comm. Jt. Res. Cent. JRC* (2016).

12. Ward, P. J. *et al. Aqueduct Floods Methodology*. *World Resources Institute* 1–28 https://www.wri.org/research/aqueduct-floods-methodology www.wri.org/publication/aqueduct-floods-methodology (2020).

13. Huizinga, J., de Moel, H. & Szewczyk, W. *Global flood depth-damage functions. Methodology and the database with guidelines*. *Joint Research Centre (JRC)* (Joint Research Centre (Seville site), 2017).

14. de Ruig, L. *et al.* An agent-based model for evaluating reforms of the National Flood Insurance Program: A benchmarked model applied to Jamaica Bay, NYC. *Risk Anal.* (2022) doi:10.1111/RISA.13905.

15. Poussin, J. K., Botzen, W. J. J. W. & Aerts, J. C. J. H. Stimulating flood damage mitigation through insurance: An assessment of the french catnat system. *Environ. Hazards* **12**, 258–277 (2013).

16. FEMA. Requirements for the Design and Certification of Dry Floodproofed Non-Residential and Mixed-Use BuildingsResidential Floodproofing-Requirements and Certification for Buildings Located in Special Flood Hazard Areas in Accordance with the National Flood Ins. (2021).

17. Dumas, P. TEMPETE XYNTHIA : RETOUR D'EXPERIENCE ET PROPOSITIONS D ' ACTION. **1**, 1–106 (2010).

18. Kousky, C. & Kunreuther, H. Addressing Affordability in the National Flood Insurance Program. *http://dx.doi.org/10.1142/S2345737614500018* **01**, 1450001 (2014).

19. Haer, T., Husby, T. G., Botzen, W. J. W. & Aerts, J. C. J. H. The safe development paradox: An agent-based model for flood risk under climate change in the European Union. *Glob. Environ. Change* **60**, 102009 (2020).

20. Duijndam, S. J. *et al.* Anticipating sea-level rise and human migration: A review of empirical evidence and avenues for future research. (2021) doi:10.1002/wcc.747.
